# Proteomic analysis in lupus mice identifies Coronin-1A as a potential biomarker for lupus nephritis

**DOI:** 10.1186/s13075-020-02236-6

**Published:** 2020-06-18

**Authors:** Orthodoxia Nicolaou, Kleitos Sokratous, Zuzanna Makowska, María Morell, Aurélie De Groof, Pauline Montigny, Andreas Hadjisavvas, Kyriaki Michailidou, Anastasis Oulas, George M. Spyrou, Christiana Demetriou, Marta E. Alarcón-Riquelme, Savvas Psarellis, Andreas Kousios, Bernard Lauwerys, Kyriacos Kyriacou

**Affiliations:** 1 Department of Electron Microscopy/Molecular Pathology, The Cyprus Institute of Neurology and Genetics, Iroon Avenue 6, Agios Dometios, 2371, P.O. Box 23462 / 1683, Nicosia, Cyprus; 2 Cyprus School of Molecular Medicine, Iroon Avenue 6, Agios Dometios, 2371, P.O. Box 23462 / 1683, Nicosia, Cyprus; 3 Bioinformatics Group, The Cyprus Institute of Neurology and Genetics, Nicosia, Cyprus; 4 Present Address: OMass Therapeutics, The Schrödinger Building, Heatley Road, The Oxford Science Park, Oxford, OX4 4GE UK; 5 Pharmaceuticals, Bayer AG, Berlin, Germany; 6 Genomic Medicine Department, Centre for Genomics and Oncological Research (GENYO), Pfizer-University of Granada-Andalusian Regional Government, Granada, Spain; 7 Institut de Recherche Expérimentale et Clinique, Université catholique de Louvain, Brussels, Belgium; 8 CHU UCL Namur, Yvoir, Belgium; 9 Biostatistics Unit, The Cyprus Institute of Neurology and Genetics, Nicosia, Cyprus; 10 Department of Primary Care and Population Health, University of Nicosia Medical School, Nicosia, Cyprus; 11 Unit of Immunology and Chronic Disease, Institute of Environmental Medicine, Karolinska Institutet, Stockholm, Sweden; 12 Department of Rheumatology, Nicosia General Hospital, Nicosia, Cyprus; 13 Renal and Transplant Centre Hammersmith Hospital Imperial College Healthcare NHS Trust, London, UK; 14 Department of Rheumatology, Cliniques Universitaires Saint-Luc, Brussels, Belgium

**Keywords:** Biomarkers, Lupus nephritis, LN, SLE, Lupus, Proteomics, Mass spectrometry

## Abstract

**Background:**

Approximately 50% of systemic lupus erythematosus (SLE) patients develop nephritis, which is among the most severe and frequent complications of the disease and a leading cause of morbidity and mortality. Despite intensive research, there are still no reliable lupus nephritis (LN) markers in clinical use that can assess renal damage and activity with a high sensitivity and specificity. To this end, the aim of this study was to identify new clinically relevant tissue-specific protein biomarkers and possible underlying molecular mechanisms associated with renal involvement in SLE, using mass spectrometry (MS)-based proteomics.

**Methods:**

Kidneys were harvested from female triple congenic B6.NZMsle1/sle2/sle3 lupus mice model, and the respective sex- and age-matched C57BL/6 control mice at 12, 24 and 36 weeks of age, representing pre-symptomatic, established and end-stage LN, respectively. Proteins were extracted from kidneys, purified, reduced, alkylated and digested by trypsin. Purified peptides were separated by liquid chromatography and analysed by high-resolution MS. Data were processed by the Progenesis QIp software, and functional annotation analysis was performed using DAVID bioinformatics resources. Immunofluorescence and multiple reaction monitoring (MRM) MS methods were used to confirm prospective biomarkers in SLE mouse strains as well as human serum samples.

**Results:**

Proteomic profiling of kidney tissues from SLE and control mice resulted in the identification of more than 3800 unique proteins. Pathway analysis revealed a number of dysregulated molecular pathways that may be mechanistically involved in renal pathology, including phagosome and proximal tubule bicarbonate reclamation pathways. Proteomic analysis supported by human transcriptomic data and pathway analysis revealed Coronin-1A, Ubiquitin-like protein ISG15, and Rho GDP-dissociation inhibitor 2, as potential LN biomarkers. These results were further validated in other SLE mouse strains using MRM-MS. Most importantly, experiments in humans showed that measurement of Coronin-1A in human sera using MRM-MS can segregate LN patients from SLE patients without nephritis with a high sensitivity (100%) and specificity (100%).

**Conclusions:**

These preliminary findings suggest that serum Coronin-1A may serve as a promising non-invasive biomarker for LN and, upon validation in larger cohorts, may be employed in the future as a screening test for renal disease in SLE patients.

## Introduction

Systemic lupus erythematosus (SLE) is a chronic inflammatory autoimmune disease that can affect almost every organ in the body, including the kidneys [1]. Renal involvement in SLE, termed lupus nephritis (LN), is one of the most frequent and severe complications of the disease. It becomes clinically apparent in about 50% of SLE patients and is considered a leading cause of morbidity and mortality in SLE [2]. Although treatment has improved over the years, the prognosis of LN remains unsatisfactory. Complete clinical remission after immunosuppressive therapy is achieved in less than 50% of patients with severe LN, and about 40% of the affected patients still present some degree of kidney impairment [3]. The failure of the conventional immunosuppressive therapy approaches to effectively treat LN makes it imperative to advance our current understanding of the pathophysiology of the disease, aiming to identify clinically valuable biomarkers and more efficacious therapeutic targets.

Currently, LN is diagnosed by assessing excreted and circulating indicators of kidney damage, such as proteinuria and serum creatinine, with supporting histological information obtained from renal biopsy. The latter, constitutes the “gold standard” for disease diagnosis, treatment and management [4]. The lack of sensitivity and specificity of conventional laboratory markers in assessing renal damage and activity, as well as the risks of invasive biopsy, imply the need for the discovery of new, non-invasive biomarkers that can be used to accurately reflect renal pathology and enable a more efficient monitoring of disease progress [5, 6].

Advances in mass spectrometry (MS) technologies and bioinformatics have enabled more comprehensive investigations of the proteome in complex biological fluids and tissues, opening new avenues for the discovery of novel biomarkers [7, 8]. Over the last few years, MS-based proteomic approaches have been extensively used to identify potential protein biomarkers that are associated with either aspects of SLE management or specific organ involvement [9].

In this study, we employed MS-based approaches to investigate alterations in the protein expression profiles of renal tissues in lupus-prone versus control mice, at different stages of disease development. The aim was to identify new, clinically relevant, tissue-specific biomarkers, as well as possible underlying molecular mechanisms associated with renal involvement in SLE.

## Material and methods

A comprehensive description of the methods is provided in the Additional file 1.

### Mice

#### Discovery set

Female B6.NZMSle123 lupus-prone and age- and sex-matched C57/Bl6 (B6) control mice were used as the discovery set. Kidneys were harvested at three time points, 12, 24 and 36 weeks of age, representing pre-symptomatic, established and end-stage LN, respectively. All mice were housed and bred under specific pathogen-free conditions. They were anaesthetized with ketamine (100 μg/g body weight) and xylazine (16 μg/g body weight) via intraperitoneal injection. After complete anaesthesia, mice were exsanguinated through transcardial perfusion using cold PBS and kidneys were then harvested. The coronal half of each kidney was snap-frozen in liquid nitrogen and preserved at − 80 °C for proteomic analysis. Experimental procedures were performed in accordance with the Ethical Committee for animal experiments of the health sciences sector, Université Catholique de Louvain, Brussels, Belgium.

#### Validation set

Four different SLE mouse strains and their age- and sex-matched controls were used as validation sets. These were the following: female B6.NZMSle123 (12 and 24 weeks), NZB/W (18 and 28 weeks), MRL/lpr (12 weeks), male BXSB. Yaa (12 and 16 weeks) lupus mice and their controls C57/Bl6, NZW, MRL/J and BXSB.B6.Yaa, respectively. All mice were housed and bred under specific pathogen-free conditions in three different centres: Catholic University of Leuven, Brussels, Belgium; BAYER Pharma AG, Berlin, Germany; Charité and Biomedical Research Center of the University of Granada, Granada, Spain. Mouse housing, handling and experimental protocols were approved by the Ethics Committee for each facility (ethical approval numbers: 2018/UCL/MD/39, G 0046/16, 12-12-14-169, respectively). Following spontaneous urine collection, all mice were anaesthetized with ketamine (100 μg/g body weight) and xylazine (16 μg/g body weight) via intraperitoneal injection. After complete anaesthesia, blood was collected through cardiac puncture and transferred into EDTA-coated tubes (Vacutainer, Beckman Dickinson). Mice were subsequently exsanguinated through transcardial perfusion using cold PBS. Kidneys were then harvested and the coronal half of each kidney was snap-frozen in liquid nitrogen and preserved at − 80 °C for proteomic analysis.

### Human samples

SLE patients and age- and sex-matched healthy controls with no history of kidney and autoimmune diseases were recruited in a single centre (The Cyprus Institute of Neurology and Genetics, Nicosia, Cyprus). LN patients included in this study were recruited in Université catholique de Louvain, Brussels, Belgium. All patients fulfilled the 1982 American College of Rheumatology revised classification criteria for the SLE diagnosis. All LN patients had biopsy-proven nephritis. Patients’ demographics are shown in Table 1. The study was approved by the Ethical Committee of Université catholique de Louvain (2014/17DEC/603) and the Cyprus National Bioethics committee (ΕΕΒΚ/ΕΠ/2015/31). All patients and controls participating in the study provided their written informed consent.

**Table 1:** Demographic data, clinical and laboratory parameters of human subjects involved in the study

Parameter	Lupus nephritis	SLE without nephritis	Healthy controls
Sum	16	18	24
Gender	Female	Female	Female
Age: Mean (SD) [range] years	33 (6.5) [24–42]	39 (8.7) [20–50]	28 (7.1) [21–50]
Race/ethnicity			
Caucasian	12 (75%)	18 (100%)	24 (100%)
Asian	1 (6.2%)	0 (0%)	0 (0%)
Other races	3 (18.7%)	0 (0%)	0 (0%)
NR	1 (6.2%)	0 (0%)	0 (0%)
Pyuria (WBC/μl) [Mean (SD), range]	41.4 (58.3) [10–166]	–	–
Proteinuria (g/L) [Mean (SD), range]	1.52 (1.2) [0.05–3.58]	–	–
Urinary creatinine (mg/dL) [Mean, (SD) range]	123.9 (66.2) [51–262]	–	–
GFR [Mean, (SD) range]	94.1 (41.5) [29–168]	–	–
C3 (mg/L) [Mean (SD), range]	0.77 (0.2) [0.5–1.25]	–	–
CRP (mg/L) [Mean (SD), range]	9 (10.9) [< 1–28]	–	–
Serum creatinine (mg/dL) [Mean (SD), range]	0.85 (0.4) [0.45–1.94]	–	–
Serum albumin (g/L) [Mean (SD), range]	22 (17.8) [3.3–44.1]	–	–
SLEDAI	8.7 (5.8) [0–21]	–	–
LN class			
II/III	1 (6.2%)	–	–
III	2 (12.5%)	–	–
III/ IV	2 (12.5%)	–	–
IV	8 (50%)	–	–
V	1 (6.2%)	–	–
NR	2 (12.5%)	–	–
Flare status			–
YES	5 (31.3%)	2 (11.1%)	–
NO	11 (68.7%)	16 (88.9%)	–
Medication			
Corticosteroids	15 (93.7%)	13 (72.2%)	–
Immunosuppressant	14 (87.5%)	10 (55.6%)	–
Anti-malarial	15 (93.7%)	17 (94.4%)	–

*NR* not reported, *GFR* glomerular filtration rate, *C3* complement component 3, *CRP* C-reactive protein, *WBC* white blood cells, *SLEDAI* SLE disease activity index

### Histology

One coronal and one transversal half of kidney from each mouse were used for histology examination. Haematoxylin and eosin (H+E) staining was performed for all samples using standard protocols, and a digital slide of all samples was obtained using a 2.0 RS Nanozoomer microscope (Hamamatsu) (see Additional file 1, Figure S6). Morphological evaluation of mouse kidneys used as a discovery set was carried out by an experienced histopathologist using a standardized set of clinical histological criteria. These criteria included the following parameters: hyper-cellularity, thrombus formation, fibrosis, cell debris and polymorphonuclear neutrophils (PMNs) infiltrate within the glomeruli, as well as tubular involvement, tubular atrophy and interstitial inflammation.

### Sample preparation for mass spectrometry analysis

#### Protein extraction from mouse kidney tissues

For each sample, 10 kidney-serial cryosections of 10-μm thickness were solubilized in lysis buffer (10 mM Tris-HCl pH 7.4, 150 mM NaCl, 1 mM EDTA, PBS) containing cOmplete Mini EDTA-free proteinase inhibitors (Roche, Germany). Following overnight acetone precipitation, protein pellets were stored at − 20 °C until further analysis.

#### Immunodepletion of serum samples

Highly abundant proteins were depleted from the human serum samples using a Human 14 Multiple Affinity Removal spin cartridge, MARS 14 (Agilent Technologies, 5188-6560, USA) according to the manufacturer’s protocol. This cartridge removes the 14 most abundant human serum proteins, namely albumin, IgG, transferrin, fibrinogen, alpha2-macroglobulin, alpha1-acid glycoprotein, antitrypsin, IgM, apolipoprotein AI, apolipoprotein AII, IgA, complement C3, transthyretin and haptoglobin.

#### Trypsin digestion and peptide purification

Following a modified FASP protocol [10], 100 μg of extracted proteins was reduced by DTT, alkylated by iodoacetamide and digested by trypsin at a 1:50 ratio, overnight at 37 °C. In the case of human samples, isotopically labeled internal standards (IS) were spiked-in in each sample. Resulting peptides were purified and desalted using solid-phase extraction cartridges (Sep-Pak tC18, Waters, Austria) and dried in a vacuum centrifuge.

### Untargeted MS proteomics

The experiments were performed on a nanoAcquity UPLC system connected to a Synapt G2Si HDMS instrument. Samples were analysed using the UDMS^e^ method [11]. The raw MS data were interpreted using the Progenesis QI for proteomics software (version 2.0, Waters, UK) against the UniProtKB/Swiss-Prot mouse reference proteome database (version 26/06/2017). (Further information is provided in the Additional file 1).

#### Data and statistical analysis

Data normalization was performed by Progenesis QIp analysis software using the “normalised to all proteins” option. Briefly, the normalization is based on the calculation of a global scaling factor which is used to normalize samples analysed multiple runs to an automatically selected reference sample. Peptide identifications were performed using the MS^e^ search identification and a peptide false discovery rate (FDR) threshold of < 1%. Identified proteins were refined using the following criteria: confidence score ≥ 5, sequence length ≥ 6 and hits ≥ 2. Protein-level relative quantitation was performed using the Hi-N approach (*N* = 3) as implemented in the Progenesis QIp. Furthermore, a variation of one-factor ANOVA calculation and an FDR approach for multiple comparisons, as implemented in the Progenesis QIp software, were used to produce a *p* value and an FDR-adjusted *p* value, or *q*-value, respectively, for every identified and quantified protein. Principal component analysis (PCA) and volcano plots were constructed using the R statistics software version R 3.5.3 (R Core Team, 2019) [12]. Data were subjected to functional enrichment and pathway analysis using the Database for Annotation, Visualization and Integrated Discovery (DAVID, version 6.8) [13].

### Targeted MRM-MS proteomics

The experiments were performed on a Waters Acquity I-Class UPLC system connected to a Waters Xevo TQD MS instrument operated on MRM mode.

#### Data analysis

Generated data were interpreted using the TargetLynx™ and Skyline software (version 4.2). Proteins of interest were quantified using external calibration curves that were constructed using standard peptides (see Additional file 1; Tables S1.1, Figure S1). In the case of human samples, quantification was performed using labeled peptides as internal standards (see Additional file 1; Tables S1.2, Figure S2). In addition, a pool of peptides of known concentration was used as a quality control (QC) and the percentage of coefficient of variation was calculated for each MRM assay (see Additional file 1; Figure S3).

#### Statistical analysis

For mouse experiments, comparisons were made using one-way of variance analysis (ANOVA) or Student’s *t-*test where appropriate. The data are presented as mean (SD). Statistical significance was established at *p* value ≤ 0.05.

For human experiments, comparisons were made using Kruskal-Wallis or Wilcoxon rank-sum non-parametric tests as appropriate. The data are presented as median and interquartile range. Correlations between CORO1A concentration and clinical characteristics were assessed using Spearman’s rank or two sample Wilcoxon rank-sum (Mann-Whitney) correlation as appropriate. Clustering analysis was performed using *K*-means-based consensus clustering, specifying two resulting groups and Euclidean distance as the similarity measure. Lastly, receiver operating curves for the ability of CORO1A concentration levels to segregate LN patients from healthy controls, as well as LN from SLE patients without nephritis, were constructed. All statistical analyses were performed using STATA version SE15 (StataCorp (2015)) and Bland-Altman plots were constructed using GraphPad Prism version 6.00 for Windows (GraphPad Software, La Jolla California USA).

### Immunofluorescence

Kidney tissue cryosections of 10-μm thickness were mildly fixed with 4% paraformaldehyde in 0.1 M phosphate buffer and blocked with 20% BSA in PBS at room temperature for 1 h. The sections were then incubated with primary antibodies against Coro1A (ab56820, 1:1000, Abcam), Arhgdib (ab181252, 1:1000, Abcam), Isg15 (PB995, 1:1000, Boster), GAPDH (sc-25778, 1:1000, Santa Cruz), at 4 °C overnight. Primary antibodies were diluted in 5% BSA and 1% Triton X in PBS. After PBS washes, secondary fluorescence antibodies, AlexaFluor 488 either coupled to anti-mouse (A-28175, 1:3000, Invitrogen) or anti-rabbit (A-11008, 1:3000, Invitrogen) antibodies, diluted in 5% BSA and 1% Triton X in PBS, were applied on slides for 1 h at room temperature. Finally, sections were washed with PBS, mounted with fluorescence mounting medium (DAKO, S3023), and examined in a ZEISS fluorescent microscope.

## Results

### Identification of differentially expressed proteins in mouse kidney tissues from control and lupus-prone mice by discovery proteomic analysis

Proteomic analysis was performed on kidneys from control (*n* = 5/time point) and lupus-prone Sle123 (*n* = 5/time point) mice at three stages of disease development, namely pre-nephritic (12 weeks), established disease (24 weeks) and end-stage renal disease (36 weeks). The penetrance of nephritis of all mice used as a discovery set was assessed by an experienced histopathologist. At 12 weeks, Sle123 mice showed normal kidney function. Overall, there is a progression in the histopathological markers at 24 and 36 weeks Sle123 mice, as shown by the mean values (Table 2), while the kidneys of wild type mice at all three time points appeared normal.

**Table 2:** Histopathology evaluation of kidneys from Sle123 discovery set

Animals	Glomerulus		Tubules	
	Hypercell/PMN/cell debris	Thrombi/fibrosis	Interstitial inflammation	Tubular involvement/atrophy
12 weeks				
**1**	**0**	**0**	**0**	**0**
**2**	**0**	**0**	**0**	**0**
**3**	**0**	**0**	**0**	**0**
**4**	**0**	**0**	**0**	**0**
**5**	**0**	**0**	**0**	**0**
Mean	**0**	**0**	**0**	**0**
24 weeks				
**1**	**3**	**1**	**1**	**1**
**2**	**1**	**0**	**0**	**0**
**3**	**1**	**0**	**1**	**0**
**4**	**3**	**1**	**3**	**1**
**5**	**0**	**0**	**1**	**2**
*Mean*	**1.6**	**0.4**	**1.2**	**0.8**
36 weeks				
**1**	**2**	**3**	**3**	**1**
**2**	**1**	**0**	**0**	**0**
**3**	**1**	**0**	**1**	**0**
**4**	**1**	**0**	**0**	**0**
**5**	**3**	**0**	**3**	**2**
Mean	**1.6**	**0.6**	**1.4**	**0.6**

Grading: 0 = normal, 1 = mild, 2 = moderate, 3 = severe. *PMN* Polymorphonuclear neutrophils

**Fig. 1:**
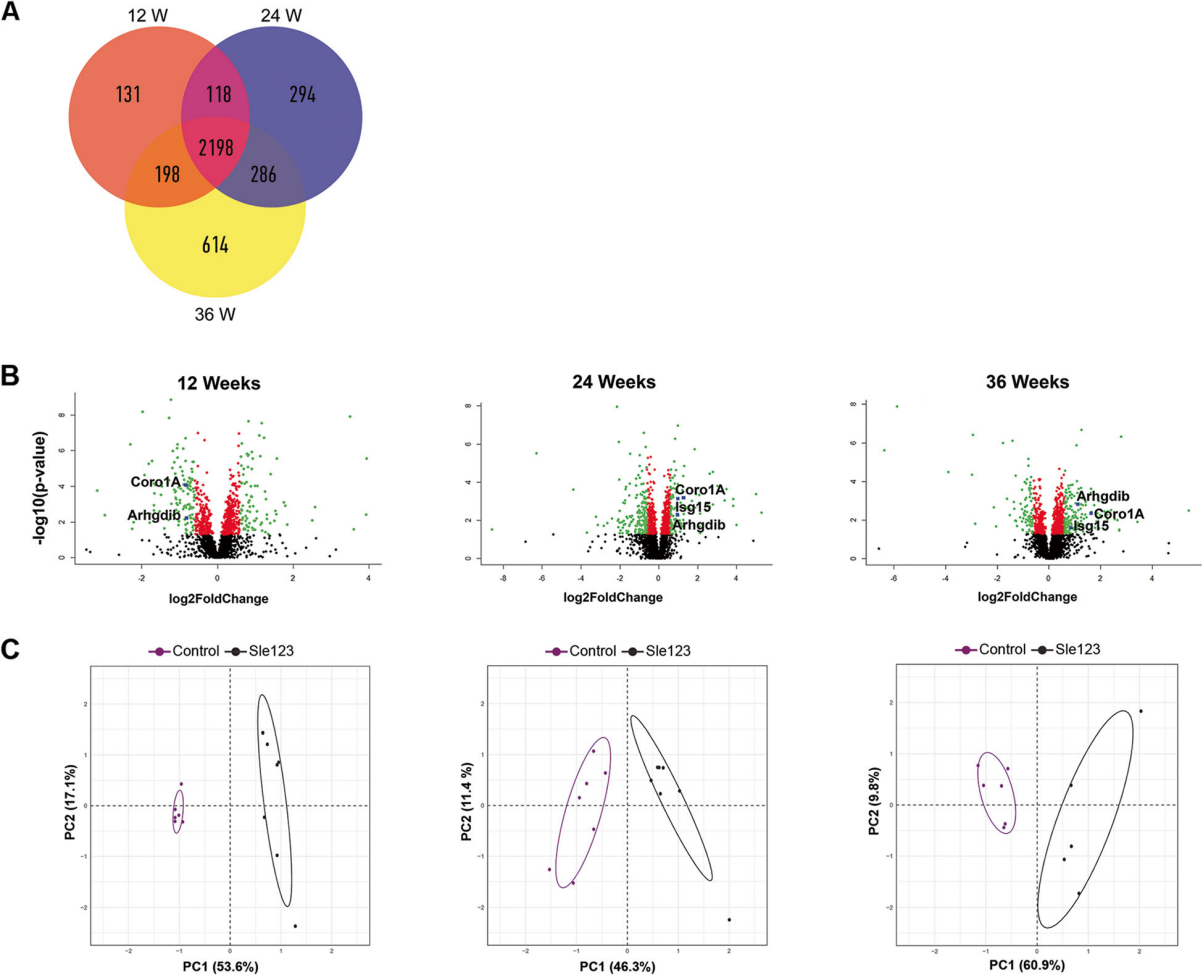
Overview of the distribution of all proteins identified by discovery proteomics among the three time points. **a** Venn diagram showing the number of shared and unique proteins identified in all samples at 12, 24 and 36 weeks. The overlapping areas represent common proteins. **b** Volcano plots of all proteins quantified by proteomic analysis comparing Sle123 (*n* = 5) and control mice (*n* = 5) at three time points, 12, 24 and 36 weeks. Plots illustrating the distributions of proteins according to their log2 fold change (*x*-axis) and −log10 *p* value (*y*-axis). Proteins showing significant differential expression, *p* value ≤ 0.05, are indicated in red. Green dots show significantly up- and downregulated proteins, with fold change ≥ 1.5 OR ≤ 0.67. The blue dots represent the selected proteins. **c** PCA score plots of protein expression patterns of all proteins with *p* value ≤ 0.05, showing clear separation of Sle123 (grey dots) and control (violet dots) mice into two clusters at all three time points. Statistical analysis was performed by one-way ANOVA test. W, Weeks, Coro1A, Coronin-1A; Arhgdib, Rho GDP-dissociation inhibitor 2; Isg15, Ubiquitin-like protein ISG15

Overall, more than 3800 unique proteins were identified among all the three time points by discovery proteomic analysis (Fig. 1a). Table 3 summarizes the number of identified proteins at each time point. More details are also given in supplementary tables (see Additional file 2; Tables S2.1 – S2.3). The distribution of the *p* values and fold changes of the whole mouse kidney proteomic profiles obtained by discovery MS-based proteomic analysis, at each time point, are illustrated in Fig. 1b as volcano plots. To enable unbiased classification of samples and detection of outliers, unsupervised PCA analysis was applied based on the 745, 806 and 899 significant differentially expressed proteins (*p* value ≤ 0.05) detected between disease and control mice at 12, 24 and 36 weeks, respectively. PCA score plots revealed a clear separation between disease and control samples into two distinct clusters based on their respective protein expression profiles at all three time points (Fig. 1c).

**Table 3:** Summary of the proteins identified by *MS-based* proteomic analysis

	12 weeks	24 weeks	36 weeks
**Total number of identified proteins**	**2645**	**2896**	**3296**
***p*value ≤ 0.05**	**745**	**806**	**899**
***p*value ≤ 0.05 AND FC ≥ 1.5 OR ≤ 0.67**	**204**	**274**	**246**
**Upregulated proteins**	**87**	**101**	**159**
**Downregulated proteins**	**117**	**173**	**87**

*FC* fold change values were scored as disease/controls

### Pathway enrichment analysis

To investigate the biological function of the identified significantly dysregulated proteins and the molecular mechanisms that may contribute to the development of LN, pathway analysis was performed. In total, 62, 63 and 66 statistically significant enriched Kyoto Encyclopedia of Genes and Genomes (KEGG) pathways (*p* value≤0.05) were identified at 12, 24 and 36 weeks of age, respectively (see Additional file 3; Table S3.1 - S3.3). The top 20 significantly enriched pathways for each time point are shown in Table 4. Next, in order to reveal pathways that may play a causative role in the development of early disease, significantly dysregulated pathways identified at each time point were compared. Of interest, 55 pathways were found to be common between all three time points, such as phagosome and proximal tubule bicarbonate reclamation and oxidative phosphorylation pathways. One pathway, namely the synthesis and degradation of ketone bodies pathway was found to be common at pre-nephritic and established disease stages. Finally, four pathways, namely galactose metabolism, adherens junction, central carbon metabolism in cancer and glucagon signaling pathways, were identified to be common at established and end-stage disease stages.

**Table 4:** Top 20 significantly enriched KEGG pathways of differentially expressed proteins identified by discovery proteomics

KEGG pathway	KEGG ID	Hits	***p*** value	FDR
**12 weeks**				
Metabolic pathways	mmu01100	483	1.45E−62	1.92E−61
Biosynthesis of antibiotics	mmu01130	144	4.13E−52	5.47E−51
Carbon metabolism	mmu01200	85	6.32E−35	8.38E−34
Oxidative phosphorylation	mmu00190	94	4.13E−34	5.48E−33
Parkinson’s disease	mmu05012	91	4.78E−28	6.35E−27
Alzheimer’s disease	mmu05010	94	8.33E−23	1.11E−21
Ribosome	mmu03010	82	2.94E−22	3.90E−21
Huntington’s disease	mmu05016	97	2.92E−20	3.88E−19
Valine, leucine and isoleucine degradation	mmu00280	43	1.66E−19	2.20E−18
Glycolysis/gluconeogenesis	mmu00010	47	1.46E−18	1.93E−17
Pyruvate metabolism	mmu00620	33	8.28E−17	1.44E−15
Non-alcoholic fatty liver disease (NAFLD)	mmu04932	75	5.96E−15	7.95E−14
Propanoate metabolism	mmu00640	25	1.82E−14	2.42E−13
Biosynthesis of amino acids	mmu01230	46	3.93E−14	5.21E−13
Fatty acid degradation	mmu00071	35	6.34E−14	8.41E−13
Peroxisome	mmu04146	48	1.10E−13	1.46E−12
Citrate cycle (TCA cycle)	mmu00020	27	1.15E−13	1.53E−12
Proteasome	mmu03050	33	1.18E−13	1.56E−12
Glutathione metabolism	mmu00480	36	1.39E−12	1.84E−11
Glycine, serine and threonine metabolism	mmu00260	28	6.95E−11	9.22E−10
**24 weeks**				
Metabolic pathways	mmu01100	490	1.27E−57	1.69E−56
Biosynthesis of antibiotics	mmu01130	144	4.10E−49	5.45E−48
Carbon metabolism	mmu01200	87	3.25E−35	4.32E−34
Oxidative phosphorylation	mmu00190	97	5.63E−35	7.49E−34
Parkinson’s disease	mmu05012	96	2.40E−30	3.19E−29
Alzheimer’s disease	mmu05010	98	9.13E−24	1.21E−22
Huntington’s disease	mmu05016	102	1.41E−21	1.88E−20
Ribosome	mmu03010	82	9.71E−21	1.29E−19
Glycolysis/gluconeogenesis	mmu00010	50	1.35E−20	1.79E−19
Valine, leucine and isoleucine degradation	mmu00280	43	1.30E−18	1.72E−17
Pyruvate metabolism	mmu00620	34	2.25E−17	2.99E−16
Propanoate metabolism	mmu00640	26	1.88E−15	2.51E−14
Fatty acid degradation	mmu00071	37	3.48E−15	4.57E−14
Biosynthesis of amino acids	mmu01230	47	4.93E−14	6.55E−13
Citrate cycle (TCA cycle)	mmu00020	27	4.22E−13	5.60E−12
Proteasome	mmu03050	33	5.43E−13	7.22E−12
Non-alcoholic fatty liver disease (NAFLD)	mmu04932	73	1.26E−12	1.67E−11
Peroxisome	mmu04146	47	4.19E−12	5.57E−11
Glutathione metabolism	mmu00480	36	6.85E−12	9.10E−11
Fatty acid metabolism	mmu01212	32	5.96E−10	7.93E−09
**36 weeks**				
Metabolic pathways	mmu01100	539	3.89E−63	5.17E−62
Biosynthesis of antibiotics	mmu01130	152	3.49E−50	4.64E−49
Carbon metabolism	mmu01200	94	6.45E−39	8.58E−38
Oxidative phosphorylation	mmu00190	100	1.22E−33	1.62E−32
Parkinson’s disease	mmu05012	95	1.04E−25	1.38E−24
Ribosome	mmu03010	90	3.47E−23	4.61E−22
Alzheimer’s disease	mmu05010	101	4.65E−22	6.18E−21
Glycolysis/gluconeogenesis	mmu00010	49	1.58E−17	2.10E−16
Huntington’s disease	mmu05016	101	1.91E−17	2.54E−16
Valine, leucine and isoleucine degradation	mmu00280	43	8.57E−17	1.44E−15
Biosynthesis of amino acids	mmu01230	52	4.61E−16	5.88E−15
Pyruvate metabolism	mmu00620	34	6.83E−16	8.88E−15
Proteasome	mmu03050	36	1.35E−14	1.80E−13
Non-alcoholic fatty liver disease (NAFLD)	mmu04932	81	2.34E−14	3.11E−13
Propanoate metabolism	mmu00640	25	7.73E−13	1.03E−11
Fatty acid degradation	mmu00071	36	1.04E−12	1.39E−11
Glutathione metabolism	mmu00480	38	4.26E−12	5.66E−11
Citrate cycle (TCA cycle)	mmu00020	27	5.98E−12	7.95E−11
Peroxisome	mmu04146	49	1.09E−11	1.45E−10
Fatty acid metabolism	mmu01212	34	3.02E−10	4.01E−09

All 20 pathways are common at all three time points. The total list of significantly dysregulated pathways is found in supplementary tables, S3.1, S3.2 and S3.3. Analysis was performed using Database for Annotation, Visualization and Integrated Discovery (DAVID) bioinformatics resources

### Identification of Coro1A, Isg15 and Arhgdib proteins as potential LN biomarkers

**Fig. 2:**
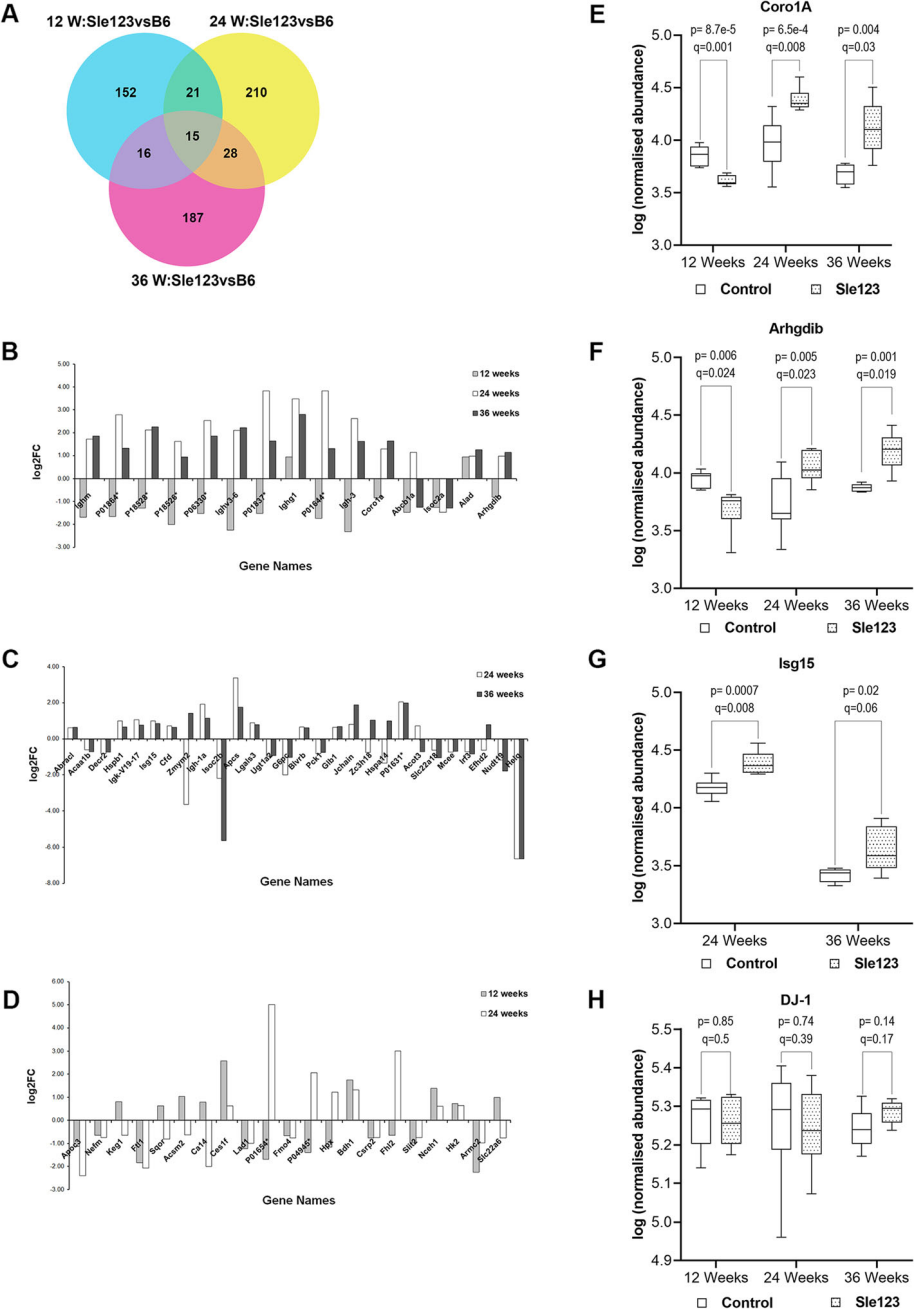
Overview of the distribution of the significant differentially expressed proteins among the three time points. **a** Venn diagram indicating the number of shared and unique proteins in Sle123 compared to control mice at 12, 24 and 36 weeks. The overlapping areas represent the common proteins. **b**–**d** Bar charts showing the fold change status of the common proteins among all three time points, 24 versus 36 weeks and 12 versus 24 weeks, respectively. The horizontal axis represents the gene symbol of each protein, and the vertical axis represents the log2 fold change (FC). Differentially expressed proteins identified by discovery proteomics at 12, 24 and 36 weeks with fold change ≥ 1.5, OR ≤ 0.67 and *p* value ≤ 0.05, were used for this analysis. Fold change values were scored as disease/controls. FC status indicates differences in the normalized protein abundances between cases and controls. **e**–**g** Whiskers plots showing the normalized protein abundances between cases and controls at the three different time points for the target proteins, Coro1A, Arhgdib and Isg15, respectively. The *p* values and *q* values as calculated by Progenesis QIp are also shown. **h** Whiskers plot showing the normalized protein abundances between cases and controls at the different time points for the Dj-1 protein used for normalization purposes. Asterisks denote UniProtKB accession numbers of proteins without available gene symbols. P01864*, Ig gamma-2A chain C region secreted form; P18528*, Ig heavy chain V region 6.96; P18526*, Ig heavy chain V region 345; P06330*, Ig heavy chain V region AC38 205.12; P01837*, Ig kappa chain C region; P01644*, Ig kappa chain V-V region HP R16.7; P01631*, Ig kappa chain V-II region 26-10; P04945*, Ig kappa chain V-VI region NQ2-6.1. W, Weeks, C57/Bl6 (B6), FC, fold change; Coro1A, Coronin-1A; Arhgdib, Rho GDP-dissociation inhibitor 2; Isg15, Ubiquitin-like protein ISG15, DJ-1; Protein/nucleic acid deglycase

Comparison of significantly altered proteins (*p* value ≤0.05, fold change ≥1.5), between the three disease stages revealed 15 common proteins (*q* value < 0.05) (Fig. 2a), of which the majority were immunoglobulins, to be upregulated at established and end-stage renal disease stages, as expected (Fig. 2b). Two proteins, namely Coronin-1A (Coro1A) and Rho GDP-dissociation inhibitor 2 (Arhgdib), were of great interest as they were found to be common at all three time points and, more importantly, were already dysregulated at pre-symptomatic stages. In particular, both proteins were found to be significantly downregulated at 12 weeks (*p* value ≤ 0.05, *q*-value ≤ 0.05, fold change ≥ 1.5), prior to the manifestation of clinical symptoms that begin at about 20 weeks in Sle123 mouse model [14], and significantly upregulated at disease active stages (*p* value ≤ 0.05, *q* value ≤ 0.05, fold change ≥ 1.5). Of note, Coro1A appears to be involved in the phagosome pathway, which was found to be dysregulated in SLE mice by pathway analysis. In addition, 28 proteins were observed to be altered at 24 and 36 weeks along with renal disease activity (Fig. 2c), while 21 proteins were common at 12 and 24 weeks (Fig. 2d). Further comparison with transcriptomic studies on human kidney biopsies of LN patients and controls performed by our PRECISESADS collaborators [15], revealed 15 common proteins, including Coro1A, Arhgdib, Ubiquitin-like protein ISG15 (Isg15) and Solute carrier family 22 member 6 (Slc22a6) (see Additional file 2; Table S2.4). Finally, taking into account the results of a comprehensive pathway analysis of the mouse proteomic data combined with the human kidney transcriptomic data, as well as prevailing evidence from an extensive literature search [16–22], we earmarked three proteins, the Coro1A, Arhgdib and Isg15, as the most promising LN biomarkers for further validation. The differences of these proteins between Sle123 and control mice at the three different time points are shown in Fig. 2e–g, respectively. Coro1A and Arhgdib levels were observed to remain stable in control mice independently of the age of mice compared to Isg15 levels that were found to decrease with the age. Coro1A levels were observed to increase at 24 weeks and decrease at 36 in Sle123 mice, while Arhgdib levels increased along with the age. Figure 2h shows the levels of DJ-1 protein, a housekeeping protein, which was used for data normalization across the three different age sample sets [23].

### Validation of the most promising biomarkers

**Fig. 3:**
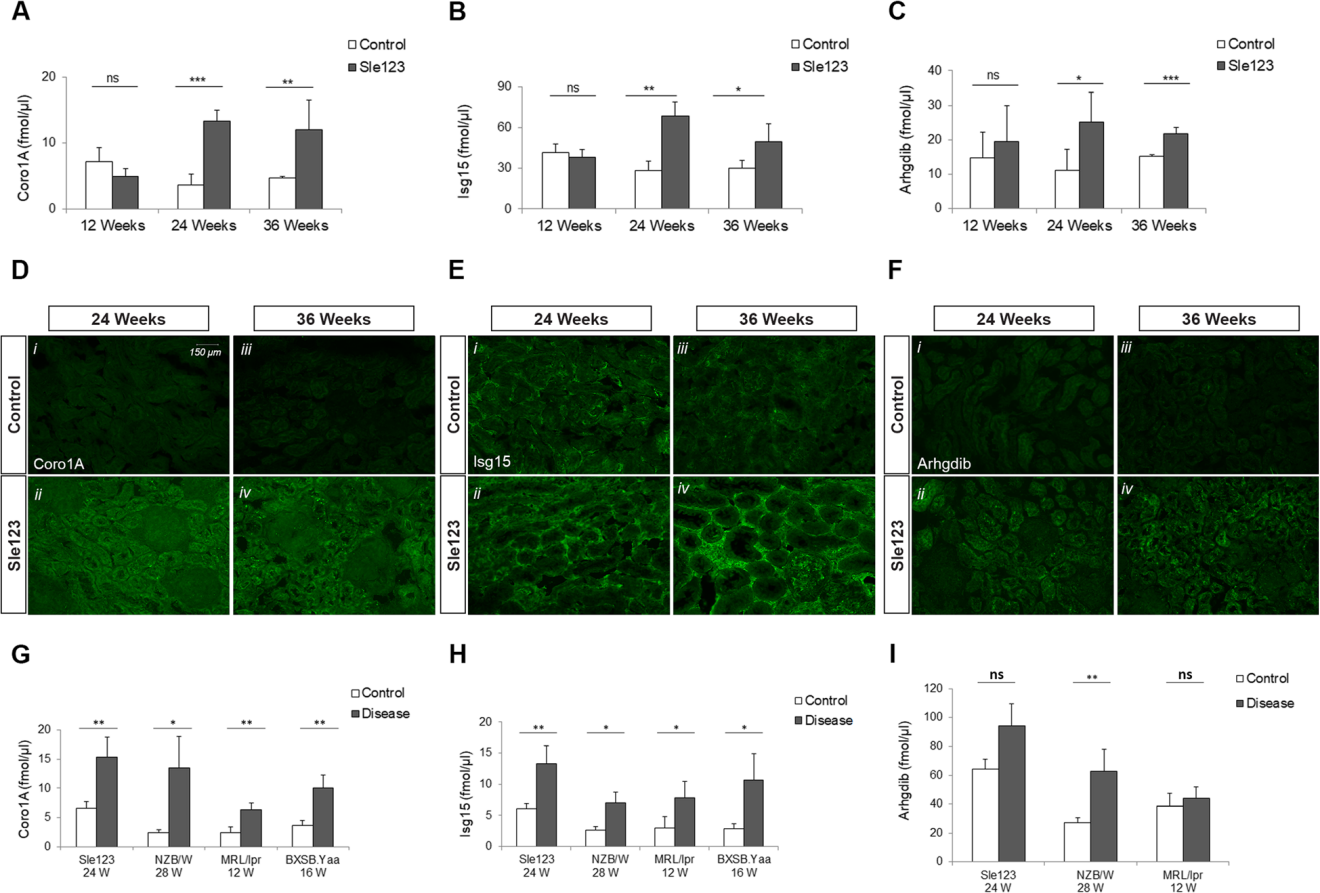
Validation of candidate protein biomarkers by MRM-MS and immunofluorescence analysis. **a**–**c** Quantification of Coro1A (**a**), Isg15 (**b**) and Arhgdib (**c**) levels in the renal tissues of Sle123 (*n* = 5) and control mice (*n* = 5) at 12, 24 and 36 weeks, by targeted MRM-MS. The data are presented as mean (SD). **d**–**f** Representative images of immunofluorescence staining for Coro1A (**d**), Isg15 (**e**) and Arhgdib (**f**) in renal tissues of Sle123 and control mice at 24 and 36 weeks. Scale bar = 150 μm. **g**–**i** Quantification of Coro1A (**g**), Isg15 (**h**) and Arhgdib (**i**) levels in the renal tissues of independent Sle123 mice cohort and three additional SLE mouse models, by targeted MRM-MS. Different time points were selected for each SLE strain based on age of disease onset. The data are presented as mean (SD) (*n* = 5 for SLE mouse strains, *n* = 3 for control mice). Statistical analysis was performed by Student’s *t* test with equal variances. Asterisks denote statistically significant differences; *p value ≤ 0.05, **p value ≤ 0.01, ***p value ≤ 0.001, ****p value ≤ 0.0001, ns, not significant. W, Weeks

The differential expression levels of the Coro1A, Isg15 and Arhgdib in kidney mouse tissues, as determined by targeted MRM-MS analysis, were found to be significantly upregulated in the kidneys of Sle123 lupus-prone compared to control mice. These differences were evident at both established and end-stage renal disease stages, having a fold difference of more than 1.5, confirming the original results generated by discovery proteomics (Fig. 3a–c). However, no significant differences were observed at pre-nephritic (12 weeks) disease stage. These observations were also confirmed by immunofluorescence analysis. Moreover, Coro1A and Arhgdib expression were found to be confined to renal tubules, while Isg15 was predominantly expressed in glomeruli and in the interstitial space (Fig. 3d–f). To further validate our results, targeted MRM-MS was also performed using another set of mouse tissues, herein referred to as the validation set. Two different time points, prior to nephritis onset and at established disease, were included in the analysis for each mouse strain based on the age of disease onset [24]. The penetrance of nephritis was assessed by H+E staining for all mice. B6.NZMSle123 (12 weeks), NZB/W (18 weeks) and BXSB. Yaa (12 weeks) showed normal kidney function with normal glomerular capillaries and tubules. B6.NZMSle123 (24 weeks), NZB/W (28 weeks), MRL/lpr (12 weeks) and BXSB. Yaa (16 weeks) mice showed loss of glomerular capillaries and development of fibrosis (see Additional file 1; Figure S6). All the respective aged- and sex-matched wild type mice for both stages showed normal glomerular capillaries and tubules (see Additional file 1; Figure S6).

Significantly increased Coro1A and Isg15 expression levels were confirmed in all SLE mouse strains, at established disease stages (Fig. 3g-h) but not at the pre-nephritic stages (see Additional file 1; Figure S4). In contrast, Arhgdib was observed to be significantly upregulated only in the kidneys of NZB/W lupus strain, at the established disease stage (Fig. 3i). It should be noted that the expression levels of this protein at pre-nephritic stages for BXSB. Yaa strain were below the detection limits of the method.

### Serum Coro1A concentration levels segregate LN patients from SLE patients without nephritis and healthy controls

**Fig. 4:**
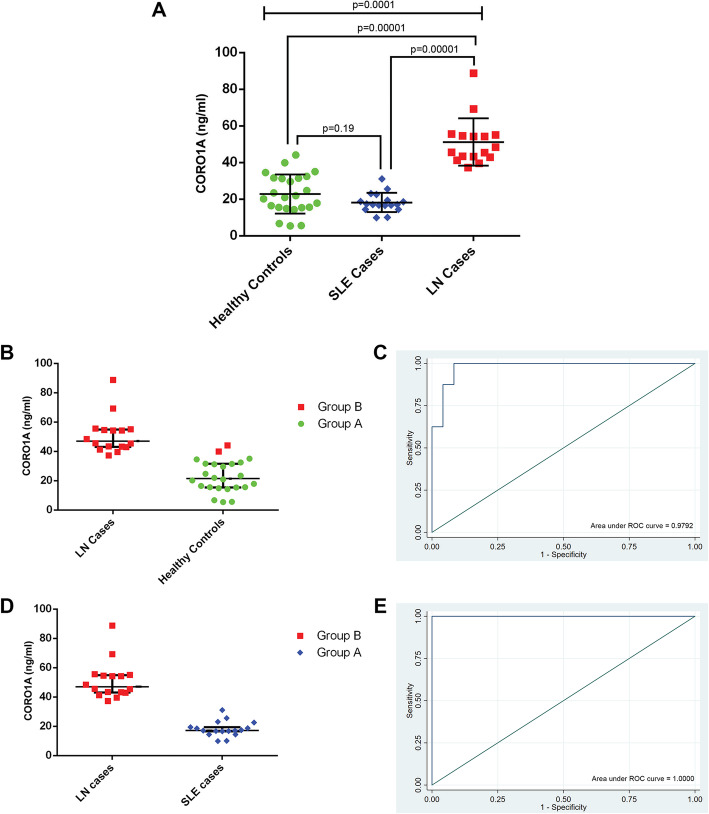
Serum CORO1A concentration levels segregate LN patients, from SLE patients without nephritis and healthy controls. Absolute quantification of Coronin-1A concentration levels in sera from LN patients (*n* = 16), SLE patients (*n* = 18) and healthy controls (*n* = 24), using targeted MRM-MS. **a** Bland-Altman plot comparing CORO1A mean concentration levels between LN cases, SLE cases and healthy controls. The median serum Coro1A concentrations were determined as 21.47 (interquartile range (IQR) 15.49–31.54), 17.13 (IQR 16.5–19.47) and 47.02 (IQR 43.085–54.84) ng/ml in the serum of healthy controls (HC), SLE and LN, respectively. Statistical analysis was performed using non-parametric tests. The three-group analysis was performed using the Kruskal-Wallis rank test (*p* value = 0.0001), while the two-group analyses using the Wilcoxon rank-sum test (HC vs SLE, *p* value = 0.19; HC vs LN, *p* value < 0.00001; SLE vs LN, *p* value < 0.00001). **b** Bland-Altman plot indicating that *K*-means based consensus clustering classifies LN patients and healthy controls into two distinct groups, shown as Group B and Group A according to their serum CORO1A concentration levels. **c** Receiver-operating characteristic (ROC) curve analysis for serum CORO1A, showing high specificity (91.67%) and sensitivity (100%) of the CORO1A to correctly detect incidences of LN and controls. Area under the curve = 0.9792, 95% confidence interval = 0.94–1.00, optimal cut-off 37.24 ng/ml. **d** Bland-Altman plot indicating that *K*-means based consensus clustering classifies LN and SLE cases into two distinct groups, shown as Group B and Group A according to their serum CORO1A concentration levels. **e** ROC curve analysis for serum CORO1A, showing high specificity (100%) and sensitivity (100%) of the CORO1A to correctly detect incidences of LN and SLE cases. Area under the curve = 1.00, 95% confidence interval = 1.00–1.00, optimal cut-off 37.24 ng/ml. Vertical lines present the median and interquartile range

To investigate whether any of the three most promising biomarkers, namely Coro1A, Isg15 and Arhgdib, identified and validated in SLE mouse models appear in human sera, an MRM-MS analysis was performed using sera from 16 SLE patients with established nephritis, 18 SLE patients without nephritis and 24 aged- and sex-matched healthy controls. Only results obtained for Coro1A are further discussed as Isg15 and Arhgdib were not detected in the human sera samples used in the present study. Absolute quantification of serum Coro1A revealed significantly increased concentration levels in the serum of LN cases compared to SLE cases and healthy controls (LN vs SLE vs HC, *p* value = 0.0001; HC vs LN, *p* value< 0.00001; SLE vs LN, *p* value< 0.00001). No significant differences were observed between SLE without nephritis cases and healthy controls (HC vs SLE, *p* value = 0.19) (Fig. 4a). The levels of serum-amyloid P-component (APCS), a protein that is known to be secreted in the human serum, were also quantified. Note that no significant alterations were observed in the serum APCS levels between LN, SLE cases and healthy controls (see Additional file 1; Figure S5). Next, unsupervised consensus clustering using *K*-means was carried out for Coro1A for LN cases and healthy controls, showing clustering of LN patients and healthy controls into two distinct groups, based on their serum Coro1A concentration levels (Fig. 4b). The ability of this protein to discriminate LN cases from healthy controls was then assessed by receiver-operating characteristic (ROC) curve analysis, as shown in Fig. 4c. Coro1A showed a sensitivity of 100% and a specificity of 91.67% in classifying LN cases and healthy controls, using 37.24 ng/ml as a cut-off. The area under the ROC curve was 0.9792 (95% confidence interval = 0.94–1.00). Most importantly, unsupervised consensus clustering between LN and SLE patients without nephritis showed clustering of LN patients and SLE into two distinct groups, based on their serum Coro1A concentration levels (Fig. 4d). The ability of this protein to discriminate LN cases from SLE was then also assessed by receiver-operating characteristic (ROC) curve analysis, as shown in Fig. 4e. Coro1A showed a sensitivity of 100% and a specificity of 100% in classifying LN cases and SLE without nephritis cases, using 37.24 ng/ml as a cut-off. The area under the ROC curve was 1.00 (95% confidence interval = 1.00–1.00). Furthermore, we attempted to decipher associations between serum Coro1A concentration levels and clinical parameters, currently used in routine clinical practice to assess renal damage and disease activity. Nevertheless, no such correlations were observed (see Additional file 1; Tables S1.3 and S1.4).

## Discussion

LN is a common complication and one of the main causes of death in SLE [2], but currently its diagnosis is challenging. Application of unbiased high-throughput proteomic approaches to LN offers unique opportunities for gaining insights into disease pathogenic mechanisms and identifying new protein biomarkers that can be used for the early diagnosis and better clinical management of this disorder [25].

In this study, proteomic profiling of kidney tissues from SLE and control mice resulted in the identification of more than 3800 unique proteins and enabled us to map significant differentially expressed proteins to molecular pathways that may be mechanistically involved in renal pathology. The majority of identified differentially expressed proteins are involved in metabolic pathways, such as fatty acid degradation, glycolysis/gluconeogenesis and oxidative phosphorylation. Defects in metabolic pathways were recently proposed to play a crucial role in the activation of the immune system that drives autoimmune diseases including SLE [26, 27]. A number of pathways found to be associated with autoimmunity and SLE have also been identified, including Fc gamma R-mediated phagocytosis and phagosome pathways [28, 29].

Of interest, proximal tubule bicarbonate reclamation pathway was found to be upregulated at pre-symptomatic stage and downregulated at active disease stages. One of the main roles of the renal proximal tubules is to sustain acid-base homeostasis by reabsorption of the majority of the filtered bicarbonate and the production of new bicarbonate, contributing to the regulation of blood pH [30]. Proximal tubules are vulnerable to hypoxic, ischemic, oxidative or metabolic injury since they are greatly dependent on aerobic oxidative metabolism [31]. Different mechanisms have been proposed through which glomerulonephritis might initiate tubulointerstitial inflammation. However, histological evidence of tubulointerstitial inflammation supports that in situ immunity might trigger and sustain local inflammation, resulting in renal damage [32]. In addition, there is growing evidence supporting a direct role of renal proximal tubular epithelial cells in renal pathogenesis in lupus [33]. Therefore, disturbances in proximal tubule bicarbonate reclamation pathway may contribute directly or indirectly to LN pathogenesis at early disease stages. However, disturbances in this pathway might also be an effect of the kidneys’ compensatory mechanism rather than a cause for lupus nephritis.

Another relevant pathway is the phagosome pathway that was observed to be commonly dysregulated in all three time points. Phagocytosis is a fundamental defence mechanism of innate immunity responsible for recognition, engulfment and elimination of invading microbial pathogens, but is also essential for tissue homeostasis through the removal of apoptotic cells. This process involves particle recognition, activation of the signalling cascade for internalization, phagosome formation and phagolysosome maturation [34]. Phagosomes function as signalling scaffolds that incorporate intra-phagosomal, intra- and extra-cellular signals, regulating phagosome maturation [35]. The important role of the clearance of apoptotic material is well-established in nearly all aspects of immunity. Still, the precise alterations of this extremely delicate and complex clearance process in SLE remains elusive [28]. Recent findings in lupus MRL/lpr mouse model suggested that defects in lysosomal maturation and acidification in macrophages could cause accumulation of apoptotic material, contained in IgG immune complexes at the membrane. Prolonged exposure to nuclear autoantigens could break self-tolerance, resulting in chronic activation of intracellular innate sensors in SLE [36]. It was further postulated that since nuclear antigens accumulate also on other professional phagocytes such as neutrophils and dendritic cells, the same lysosomal defect may lead to the activation of various cell types in SLE, causing other disease manifestations [36–38]. Nevertheless, whether disturbances in the phagosome pathway have a causative role in SLE and LN or this may be a secondary effect due to increased cellular stress and upregulation of the unfolded protein response remains to be seen.

Further evaluation of the proteomic profiling results obtained in this study, led to the identification of three proteins, namely Arhgdib, Isg15 and Coro1a, as potential markers of LN. Notably, our results were further supported by transcriptomic data obtained from human kidney biopsies [15] and were validated in additional spontaneous and induced SLE mouse models. To our knowledge these proteins have not been identified previously in proteomics studies in other kidney diseases [39, 40].

Coro1A is a member of an evolutionary conserved actin-associated family of proteins, which were initially identified as important regulators of actin cytoskeleton-dependent processes, including phagocytosis, cell migration, polarization and cytokinesis [41, 42]. In mammals, Coro1A is mainly expressed in haematopoietic cells and appeared to play a critical role in lymphocyte trafficking, T cell receptor signalling, Ca2+ signalling in macrophages and modulating endothelial adhesion events [43–46]. Coronin mutations have been associated with immune deficiencies and resistance to autoimmunity both in humans and mice [16]. A nonsense-mutation in the Coro1A gene has been shown to suppress autoimmunity and disease development in SLE murine models [17]. Lastly, monoclonal antibodies against Coro1A were identified as a potential therapy for auto-inflammatory diseases and B cell malignancies, supporting the notion that Coro1A is involved in the overall regulation of the immune system [16]. In this study, we have demonstrated that Coro1A is significantly upregulated in the kidney tissues, particularly in renal tubules, of SLE mouse models compared to control mice. These results confirm previous data obtained in humans by transcriptomic studies on lupus biopsies [15]. These data suggest that intra-renal activation of immune effectors against renal tubular cells plays a determining role in disease progression. In addition, Coro1A was found to be implicated in the phagosome pathway, as shown by our results, that is known to play an important role in the clearance of apoptotic cells, as mentioned above. An ideal biomarker, apart from its biologic and pathophysiologic relevance, should be easily measurable in a readily accessible biological sample. To this end, we tried to determine the concentration of the three target proteins, identified in the kidneys of mice, in human serum of healthy controls as well as in SLE patients with or without nephritis. We showed for the first time that Coro1A is secreted in human serum and can distinguish LN patients from SLE patients without nephritis with high sensitivity and specificity. However, we were unable to correlate Coro1A levels with currently used disease clinical parameters. This discrepancy may in part be attributed to the fact that the present LN classification system focuses mainly on glomerular pathology and does not take into account the changes in the tubules, where Coro1A is localized. Taken together, our findings suggest that Coro1A may exert an important effect in renal immunopathology in lupus and may serve as a potential screening biomarker for LN. These results, however, are preliminary and further larger multicentre studies are required using well-characterized patient cohorts as well as disease control groups, to prove the utility of this protein in routine clinical practice as a potential non-invasive test for screening of renal disease in SLE.

Isg15 is a small ubiquitin-like protein activated by type I interferons (IFNs), which play a central role in innate immunity by regulating host antiviral responses either through its binding to a target protein (ISGylation), or its function as a free protein. Its unconjugated form has been mostly correlated with cytokine production and activation of immunocytes [47, 48]. Secreted Isg15 stimulates INF-γ secretion by T cells, as well as T cell-dependent proliferation of natural killer cells in B cell depleted lymphocyte cultures [49]. Additionally, it was suggested that Isg15 may serve as a potential bridge between type I (IFN-α, IFN-β) and type II (IFN-γ) IFN-mediated immune responses, which are both known to be implicated in SLE pathogenesis [20, 50]. Moreover, a strong IFΝ signature has been observed in patients with SLE, characterized by elevated expression of type I IFN regulated genes [18]. It has been postulated that activation of Janus kinase (JAK)-signal transducer and activator of transcription (STAT) 1 pathway, by either IFN-α or IFN-γ stimulation, contributes to the immunopathogenesis of LN [19, 20]. In the present study, Isg15 was observed to be upregulated in the kidney tissues, predominantly in renal interstitium and glomeruli, of SLE mouse models compared to control mice. A recent study suggested a potential involvement of Isg15 dysregulation in the pathogenesis of glomerular inflammation [21]. Therefore, we postulate that Isg15 may play an important role in renal pathology in lupus and may serve as a potential marker of kidney inflammation and degree of injury in SLE.

Arhgdib is a member of the Rho GDP dissociation inhibitors family, which are small GTP-binding proteins and act as negative regulators of the Rho family of small GTPases. In humans, Arhgdib is expressed primarily in haematopoietic tissues, mostly T and B cells [22, 51] and is known to be implicated in a variety of cellular processes, including cytoskeletal organization, cell signaling and proliferation, as well as apoptosis [52–54]. However, there is limited information available about the intracellular regulatory function of this protein. In a recent study, autoantibodies specific to Arhgdib were detected in the sera of a large subset of SLE patients. It was suggested that these antibodies can induce important responses in T lymphocytes, such as autophagy and Rho GTPase cytoskeleton remodelling [22]. In SLE, prolonged exposure to anti-Arhgdib autoantibodies might result in autophagy of resistant T cell clones, contributing to disease pathogenesis. Studies in both lupus-prone and SLE patients have implicated autophagy in LN [22]. In the present study, Arhgdib protein was found to be upregulated in the kidneys, primarily in renal tubules, of lupus-prone mice compared to control mice at established and end-stage renal disease. Taken together, these findings suggest that Arhgdib plays an important role in renal disease pathogenesis in lupus.

## Conclusion

Overall, MS-based proteomic analysis enabled us to detect a significant number of differentially expressed proteins in the kidneys of SLE mice and identify underlying molecular mechanisms involved in renal pathology. Three proteins, namely Arhgdib, Isg15 and Coro1A, which play key roles in both immune and renal responses, have been selected as potential candidate biomarkers of LN. Further functional studies that will advance our understanding on their precise role in LN are required. In addition, we demonstrated for the first time that serum levels of Coro1A can be used as a marker, able to distinguish LN from SLE patients with high specificity and sensitivity. These promising data need to be further explored and validated. Quantification of Coro1A in sera of larger, well-annotated patient cohorts will provide further evidence for its role in renal disease, as well as its value as a screening LN biomarker.

## Supplementary information

**Additional file 1:** Supplemental methods, Tables and Figures. Supplemental methods: Further details about sample preparation for mass spectrometry analysis, untargeted LC-MS/MS proteomics and targeted proteomic analysis. **Table S1.1:** MRM parameters for the synthesized peptides of the selected mouse kidney target proteins. **Table S1.2:** MRM parameters for the synthesized peptides of human serum target and immunodepleted proteins. **Table S1.3:** Spearman’s correlation analysis between CORO1A serum levels and clinical parameters in LN patients. **Table S1.4:** Two sample Wilcoxon rank-sum (Mann-Whitney) correlation analysis between CORO1A serum levels and clinical parameters in LN patients. **Figure S1:** Representative chromatograms of target peptides used in the targeted MRM-MS proteomic analysis for the mouse experiments. **Figure S2:** Representative chromatograms of target peptides used in the targeted MRM-MS proteomic analysis for the human experiments. **Figure S3:** Coefficient of variation of MRM assays for both mouse and human experiments. **Figure S4**: MRM-MS analysis of candidate protein biomarkers in different SLE mouse strains at pre-nephritic stage. **Figure S5**: Concentration levels of serum Amyloid P-component protein in SLE, LN patients and healthy controls. Figure S6: Representative images of H + E staining of the validation set.

**Additional file 2. **Discovery proteomics data (accession number, protein names, *p* value, q-value, fold change status of identified proteins). List of common proteins between mouse kidney discovery proteomics and human kidney transcriptomics. **Table S2.1**: List of all proteins quantified by discovery proteomics in the kidneys of Sle123 compared to control mice at 12 weeks. **Table S2.2**: List of all proteins quantified by discovery proteomics in the kidneys of Sle123 compared to control mice at 24 weeks. **Table S2.3**: List of all proteins quantified by discovery proteomics in the kidneys of Sle123 compared to control mice at 36 weeks. **Table S2.4**: List of 15 common proteins between mouse kidney discovery proteomics and human kidney transcriptomics.

**Additional file 3.** List of significantly enriched KEGG pathways identified by pathway analysis of discovery proteomic data at each time. **Table S3.1**: List of significantly enriched KEGG pathways at 12 weeks. **Table S3.2**: List of significantly enriched KEGG pathways at 24 weeks. **Table S3.3**: List of significantly enriched KEGG pathways at 36 weeks.

## Data Availability

The datasets used and/or analysed during the current study are available from the corresponding author on reasonable request.

## Abbreviations

APCS: Serum-amyloid P-component
Arhgdib: Rho GDP-dissociation inhibitor 2
Coro1A: Coronin-1A
DAVID: Database for Annotation, Visualization and Integrated Discovery
FDR: False discovery rate
IS: Internal standards
Isg15: Ubiquitin-like protein ISG15
KEGG: Kyoto Encyclopedia of Genes and Genomes
LN: Lupus nephritis
MRM: Multiple reaction monitoring
MS: Mass spectrometry
PCA: Principal component analysis
PMN: Polymorphonuclear neutrophils
QC: Quality control
ROC: Receiver-operating characteristic
SLE: Systemic lupus erythematosus
