# Supplementary material for: Proteomic analysis in lupus mice identifies Coronin-1A as a potential biomarker for lupus nephritis
Source: Arthritis Res Ther. 2020 Jun 18;22:147. doi: 10.1186/s13075-020-02236-6 (PMC7301983; doi:10.1186/s13075-020-02236-6)
Supplement: Supplementary file 1 — Additional file 1: Supplemental methods, Tables and Figures. Supplemental methods: Further details about sample preparation for mass spectrometry analysis, untargeted LC-MS/MS proteomics and targeted proteomic analysis. Table S1.1: MRM parameters for the synthesized peptides of the selected mouse kidney target proteins. Table S1.2: MRM parameters for the synthesized peptides of human serum target and immunodepleted proteins. Table S1.3: Spearman’s correlation analysis between CORO1A serum levels and clinical parameters in LN patients. Table S1.4: Two sample Wilcoxon rank-sum (Mann-Whitney) correlation analysis between CORO1A serum levels and clinical parameters in LN patients. Figure S1: Representative chromatograms of target peptides used in the targeted MRM-MS proteomic analysis for the mouse experiments. Figure S2: Representative chromatograms of target peptides used in the targeted MRM-MS proteomic analysis for the human experiments. Figure S3: Coefficient of variation of MRM assays for both mouse and human experiments. Figure S4: MRM-MS analysis of candidate protein biomarkers in different SLE mouse strains at pre-nephritic stage. Figure S5: Concentration levels of serum Amyloid P-component protein in SLE, LN patients and healthy controls. Figure S6: Representative images of H + E staining of the validation set. [file 13075_2020_2236_MOESM1_ESM.docx]

# Supplementary file 1

## **Supplementary Methods**

## Sample preparation for mass spectrometry analysis

### Protein extraction from mouse kidney tissues

For each sample, 10 kidney serial cryosections of 10μm thickness, cut with a cryostat (Bright’s, UK) were solubilized in lysis buffer (10mM Tris-HCl pH 7.4, 150mM NaCl, 1mM EDTA, PBS) containing cOmplete Mini EDTA-free proteinase inhibitors (Roche, Germany). The samples were incubated in ice for 30 min, followed by sonication and acetone precipitation. Following overnight precipitation, samples were centrifuged at 13,000×g for 15 minutes at 4^o^C, the supernatant was discarded and the remaining protein pellet was air dried. The protein pellets were stored at -20^o^C until further analysis.

### Immunodepletion of serum samples

Highly abundant proteins were depleted from the human serum samples using a Human 14 Multiple Affinity Removal spin cartridge, MARS 14 (Agilent Technologies, 5188-6560, USA). This cartridge removes the 14 most abundant human serum proteins, namely albumin, IgG, transferrin, fibrinogen, alpha2-macroglobulin, alpha1-acid glycoprotein, antitrypsin, IgM, apolipoprotein AI, apolipoprotein AII, IgA, complement C3, transthyretin and haptoglobin. Briefly, 0.45 ml spin cartridge was used to deplete 10 μl sera from each sample, twice (in total 20 μl of serum /per sample). Two different buffers, A and B, were required for this process (Agilent Technologies, 5188-6560, USA). The pH 7.4 phosphate salt-containing buffer A was used for loading, washing and re-equilibration steps, while buffer B, a low pH urea buffer, for eluting the high abundance proteins, bound on the cartridge. In brief, 10 μl of serum sample were first diluted 20 X with buffer A and transferred to 0.22 μm spin-filters to remove particulates. Upon centrifugation at 13000 x g for two minutes, samples were applied onto the cartridge. The cartridge was centrifuged for 1 minute at 100 x g, followed by incubation at room temperature for 5 minutes. Cartridge was then washed with 400 μl buffer A and centrifuged for 2.5 minutes at 100 x g, twice. The flow-through fractions (F1 and F2) containing the low abundance proteins were collected, precipitated overnight in cold acetone and pelleted by centrifugation at 13,000×g for 15 minutes at 4^o^C. The supernatant was then discarded and the remaining protein pellet was air dried and stored at -80^o^C until further analysis.

### Trypsin digestion and peptide purification

Protein pellets were then re-dissolved in 8M urea/50mM NH4HCO3 buffer and concentration was determined by BCA assay (Thermo Fisher Scientific, UK). Following a modified FASP protocol [1], 100 µg of extracted proteins were added on centrifugation filter (NanoSep Omega, 30 kDa MWCO), reduced by 8 mM DTT (Thermo Fisher Scientific, UK) for 30 minutes at 56^o^C, alkylated by 50 mM IAM (Thermo Fisher Scientific, UK) for 15 minutes at room temperature in dark and digested by trypsin (Trypsin recombinant, proteomics grade, Roche, Germany) at a 1:50 ratio. In the case of human samples, 10 μL of isotopically labelled internal standards (IS), 0.2 pmol/μl IS of target proteins and 1 pmol/μl IS of immunodepleted peptides final concentration, were spiked-in, in each sample. Resulting peptides were purified and desalted using a C18 solid phase extraction cartridge (Sep-Pak tC18, Waters, Austria) and dried in a vacuum centrifuge.

## Untargeted LC-MS/MS proteomics

Purified peptides were resuspended in buffer (95% water, 5% acetonitrile, 0.1% formic acid) to a final protein concentration of 0.2 μg/μl, transferred to autosampler vials and 2 μl were loaded onto C18 column (nanoAcquity CSH C18, 75 μm ID x 150 mm, 1.7 μm, Waters, UK). Samples were eluted by linear gradient from 5 to 40% of mobile phase B (0.1% formic acid in acetonitrile) over 175 minutes. Peptides were analysed on a Synapt G2-Si HDMS instrument operated on ion mobility mode using the UDMS^e^ [2] method. A minimum of three biological replicates and two technical replicates were used for the analysis.

## Targeted proteomic analyses

Proteins of interest identified in the discovery proteomic experiments were further cross validated, using LC-MRM-MS. For each target protein, two associated peptides were selected based on the following criteria: a) identified in the discovery phase by Progenesis QIp software with probability greater than 90%, b) completely digested by trypsin, c) excluding peptides with M, RP, KP amino acid residues and glycosylation site prone to artefactual or post-translational modifications, and d) 7-25 amino acid residues. In addition, SRMAtlas [3] and Skyline (version 4.2) [4] were also employed in order to select the most appropriate peptides and MRM transitions for the identification and quantification of the proteins of interest. For verification experiments crude purity peptide standards were purchased from JPT peptide technologies (Berlin, Germany), while in the case of validation experiments, in both mice and humans, high purity peptide standards (>99%) were purchased from Pepscan (Lelystad, The Netherlands). In this manner, a multiplex panel capable of detecting the peptides of interest was designed. Dried peptides derived from kidney mice tissue or human sera were re-suspended with 0.1% formic acid in 5% acetonitrile. Diluted peptides were then transferred to autosampler vials and 10 μl were loaded onto C18 column (Acquity UPLC CSH^TM^ C18, 1mm x 150 mm, 1.7 μm, Waters, UK). The chromatographic separation of peptides was performed using a UPLC system (Acquity I-Class, Waters, UK) and the eluted peptides were analysed on a Waters Xevo TQD MS system equipped with the standard ESI source and operated on MRM mode.

# References

1. Wisniewski JR, Zougman A, Nagaraj N, et al. Universal sample preparation method for proteome analysis. Nat Methods. 2009;6(5):359-62.

2. Distler U, Kuharev J, Navarro P, et al. Drift time-specific collision energies enable deep-coverage data-independent acquisition proteomics. Nat Methods. 2014;11(2):167-70.

3. Picotti P, Rinner O, Stallmach R, et al. High-throughput generation of selected reaction-monitoring assays for proteins and proteomes. Nat Methods. 2010;7(1):43-6.

4. MacLean B, Tomazela DM, Shulman N, et al. Skyline: an open source document editor for creating and analyzing targeted proteomics experiments. Bioinformatics. 2010;26(7):966-8.

Table S1.1: MRM parameters for the synthesized peptides of the selected mouse kidney target proteins

| **Accession Number** | **Gene**  **Name** | **Peptide sequence** | **MW** | **Precursor m/z (Da)** | **Products m/z (Da)** | **Retention Time (min)** | **Collision Energy (V)** | **Cone Voltage (V)** |
| --- | --- | --- | --- | --- | --- | --- | --- | --- |
| P31146 | Coro1a | **DAGPLLISLK** | 1026.23 | 514.1^2+^ | 392.4  [y7 ++] | 18.7 | 18 | 28 |
|  |  |  |  |  | 420.9  [y8 ++] |  |  |  |
|  |  |  |  |  | 840.8  [y8 +] |  |  |  |
|  |  | ADQZYEDVR | 1155.19 | 578.4^2+^ | 485.4  [y7++] | 6.7 | 20 | 32 |
|  |  |  |  |  | 681.4  [y5 +] |  |  |  |
|  |  |  |  |  | 841.6  [y6 +] |  |  |  |
|  |  | VSQTTWDSGFZAVNPK | 1796.95 | 899.3^2+^ | 979.7  [y9 +] | 12.7 | 34 | 50 |
|  |  |  |  |  | 1094.8  [y10 +] |  |  |  |
|  |  |  |  |  | 1281.1  [y11 +] |  |  |  |
| Q61599 | Arhgdib | **YVQHTYR** | 966.05 | 483.8^2+^ | 439.2  [y3 +] | 3.8 | 20 | 40 |
|  |  |  |  |  | 576.3  [y4 +] |  |  |  |
|  |  |  |  |  | 704.3  [y5 +] |  |  |  |
|  |  | ATFMVGSYGPRPEEYEFLTPVEEAPK | 2946 | 982.8^3+^ | 1044.6  [y18 ++] | 20.5 | 34 | 35 |
|  |  |  |  |  | 1198.4  [y21 ++] |  |  |  |
|  |  |  |  |  | 1248  [y22 ++] |  |  |  |
| Q64339 | Isg15 | **IGVPAFQQR** | 1015.16 | 508.5^2+^ | 373.7  [y6 ++] | 10.5 | 16 | 28 |
|  |  |  |  |  | 423.2  [y7 ++] |  |  |  |
|  |  |  |  |  | 746.4  [y6 ++] |  |  |  |
|  |  | GHSNIYEVFLTQTVDTLK | 2065.28 | 689.5^3+^ | 631.7  [b11 ++] | 21 | 16 | 30 |
|  |  |  |  |  | 905.4  [y8 +] |  |  |  |

*Peptides used for quantitation are indicated in bold.*

Table S1.2: MRM parameters for the synthesized peptides of human serum target and immunodepleted proteins

| **Accession**  **Number** | **Gene**  **Name** | **Peptide Sequence** | **MW**  **(Da)** | **Precursor**  **m/z (Da)** | **Products**  **m/z (Da)** | **Retention**  **Time (min)** | **Collision**  **Energy (V)** | **Cone**  **Voltage (V)** |
| --- | --- | --- | --- | --- | --- | --- | --- | --- |
| **TARGET PROTEINS** | | | | | | | | |
| P31146 | CORO1A | DAGPLLISL**K*** | 1034.3 | 517.8^2+^ | 396.3  [y7++] | 16.9 | 16 | 35 |
|  |  |  |  |  | 424.8  [y8++] |  |  |  |
|  |  |  |  |  | 848.6  [y8+] |  |  |  |
|  |  | DAGPLLISLK | 1026.3 | 513.8^2+^ | 392.3  [y7++] | 16.9 | 16 | 35 |
|  |  |  |  |  | 420.8  [y8++] |  |  |  |
|  |  |  |  |  | 840.6  [y8+] |  |  |  |
|  |  | ADQZYEDVR | 1155.2 | 578.2^2+^ | 485.2  [y7++] | 7.2 | 22 | 35 |
|  |  |  |  |  | 681.3  [y5+] |  |  |  |
|  |  |  |  |  | 841.3  [y6+] |  |  |  |
| P02743 | APCS | VGEYSLYIG**R*** | 1166.3 | 583.8^2+^ | 242.1  [y2+] | 13.5 | 22 | 35 |
|  |  |  |  |  | 518.3  [y4+] |  |  |  |
|  |  |  |  |  | 718.4  [y6+] |  |  |  |
|  |  | VGEYSLYIGR | 1156.3 | 578.8^2+^ | 232.1  [y2+] | 13.5 | 22 | 35 |
|  |  |  |  |  | 508.3  [y4+] |  |  |  |
|  |  |  |  |  | 708.4  [y6+] |  |  |  |
|  |  | IVLGQEQDSYGGK | 1393.5 | 697.3^2+^ | 591.3  [y11++] | 9.9 | 24 | 35 |
|  |  |  |  |  | 1068.5  [y10+] |  |  |  |
|  |  |  |  |  | 1181.5  [y11+] |  |  |  |
| **IMMUNODEPLETED PROTEINS** | | | | | | | | |
| P02768 | ALB | FQNALLV**R*** | 970.2 | 485.8^2+^ | 276.1  [b2+] | 11.8 | 15 | 35 |
|  |  |  |  |  | 510.4  [y4+] |  |  |  |
|  |  |  |  |  | 695.4  [y6+] |  |  |  |
|  |  | FQNALLVR | 960.2 | 480.8 ++ | 276.1  [b2+] | 11.8 | 15 | 35 |
|  |  |  |  |  | 500.4  [y4+] |  |  |  |
|  |  |  |  |  | 685.4  [y6+] |  |  |  |
| P02787 | TF | DGAGDVAFV**K*** | 986.1 | 493.7++ | 244.1  [b3+] | 10.9 | 17 | 35 |
|  |  |  |  |  | 472.3  [y4+] |  |  |  |
|  |  |  |  |  | 743.4  [y7+] |  |  |  |
|  |  | DGAGDVAFVK | 978.1 | 489.7 ++ | 244.1  [b3+] | 10.9 | 17 | 35 |
|  |  |  |  |  | 464.3  [y4+] |  |  |  |
|  |  |  |  |  | 735.4  [y7+] |  |  |  |
| P01009 | SERPINA1 | SVLGQLGIT**K*** | 1023.2 | 512.3 ++ | 187.1  [b2+] | 14.4 | 16 | 35 |
|  |  |  |  |  | 419.3  [y8++] |  |  |  |
|  |  |  |  |  | 837.5  [y8+] |  |  |  |
|  |  | SVLGQLGITK | 1015.2 | 508.3 ++ | 187.1  [b2+] | 14.4 | 16 | 35 |
|  |  |  |  |  | 415.3  [y8++] |  |  |  |
|  |  |  |  |  | 829.5  [y8+] |  |  |  |
| P00738 | HP | TEGDGVYTLNNE**K*** | 1455.5 | 724.3 ++ | 889.4  [y7+] | 9.5 | 26 | 35 |
|  |  |  |  |  | 1045.5  [y9+] |  |  |  |
|  |  |  |  |  | 1217.6  [y11+] |  |  |  |
|  |  | TEGDGVYTLNNEK | 1447.5 | 720.3 ++ | 881.4  [y7+] | 9.5 | 26 | 35 |
|  |  |  |  |  | 1037.5  [y9+] |  |  |  |
|  |  |  |  |  | 1209.6  [y11+] |  |  |  |

** Pre-labeled peptides either on Arginine (R) or Lysine (K) amino acid.*

Table S1.3: Spearman’s correlation analysis between CORO1A serum levels and clinical parameters in LN patients

| Clinical parameter | # of obs | Rho (ρ) | p-value |
| --- | --- | --- | --- |
| Pyuria (WBC/μl) | 13 | 0.02 | 0.95 |
| Proteinuria (g/L) | 15 | 0.21 | 0.45 |
| Urinary creatinine (mg/dL) | 15 | 0.18 | 0.52 |
| GFR (mL/min) | 15 | 0.05 | 0.85 |
| C3 (mg/L) | 13 | 0.26 | 0.38 |
| CRP (mg/L) | 14 | -0.17 | 0.55 |
| Serum creatinine (mg/dL) | 15 | -0.11 | 0.70 |
| Serum albumin (g/L) | 13 | -0.05 | 0.86 |
| SLEDAI | 16 | -0.24 | 0.37 |
| LN class | 14 | -0.05 | 0.86 |
| BMI (kg/m^2^) | 15 | -0.12 | 0.66 |

Data are presented as correlation coefficient Rho (ρ). GFR: glomerular filtration rate; C3: complement component 3; CRP: C-reactive protein; WBC: white blood cells; SLEDAI: SLE disease activity index; BMI: Body mass index; obs: observations.

Table S1.4: Two sample Wilcoxon rank-sum (Mann-Whitney) correlation analysis between CORO1A serum levels and clinical parameters in LN patients

| Clinical parameter | Median | p-value |
| --- | --- | --- |
| Flare status: Yes | 48.41 | 0.87 |
| Flare status: No | 55.1 |  |
| Smoking: Yes | 54.58 | 0.47 |
| Smoking: No | 47.02 |  |

## Supplementary Figures

## Figure Legends

## Figure S1: Representative chromatograms of target peptides used in the targeted MRM-MS proteomic analysis for the mouse experiments.

Chromatograms of all peptides used for detecting target proteins in the mouse experiments. (A) Coronin-1A (Coro1a). (B) Rho GDP-dissociation inhibitor 2 (Arhgdib). (C) Ubiquitin-like protein ISG15 (Isg15). Minimum two peptides for each protein were included in the analysis. Peptide sequence, retention time, M/Z and peak intensities are shown for each peptide.

## Figure S2: Representative chromatograms of target peptides used in the targeted MRM-MS proteomic analysis for the human experiments.

Chromatograms of all peptides used for detecting target proteins in the human experiments. (A) Coronin-1A (Coro1A). (B) Serum-amyloid P-component (APCS). (C) Serum albumin (ALB). (D) Serotransferrin (TF). (E) Alpha-1-antitrypsin (SERPINA1), (F) Haptoglobin (HP). Minimum two peptides for each target protein were included in the analysis. Peptide sequence, retention time, M/Z and peak intensities are shown for each peptide.

## Figure S3: Coefficient of variation of MRM assays for both mouse and human experiments.

A pool of peptides of known concentration was used as a quality standard (QC) and the percentage of coefficient of variation was calculated for each MRM assay. (A) Bar chart showing the percentage of coefficient of variation for the MRM assay in the Sle123 mouse model used as a discovery set. (B-E) Bar charts indicating the percentage of coefficient of variation for the MRM assays in the Sle123 mouse model used as a validation set, NZB/W,MRL/lpr and BXSB.Yaa mouse models, respectively. (F) Percentage of coefficient of variation of MRM assay for human experiments. CV, Coefficient of Variation, QC, Quality Standard.

**Figure S4: MRM-MS analysis of candidate protein biomarkers in different SLE mouse strains at pre-nephritic stage.**

(A-C) Targeted MRM-MS analysis of (A) Coronin-1A (Coro1a), (B) Ubiquitin-like protein ISG15 (Isg15), and (C) Rho GDP-dissociation inhibitor 2 (Arhgdib) in renal tissues from Sle123 independent cohort, NZB/W and BXYaa lupus-prone and their respective control mice at 12, 18 and 12 weeks of age, respectively. The data are presented as mean (SD) (n=5 for SLE mouse strains, n=3 for control mice). No statistically significant difference was observed in the protein expression levels of both proteins at early disease stages, prior to nephritis onset, in all three SLE strains. Arhgdib levels in BXYaa lupus-prone were below detection limits. Statistical analysis was performed by Student’s t-test, equal variances. P-value of 0.05 was considered as statistically significant differential expression. W, Weeks.

**Figure S5: Concentration levels of serum Amyloid P-component protein in SLE, LN patients and healthy controls.**

Absolute quantification of serum Amyloid P-component (APCS) concentration levels in sera from LN patients (n=16), SLE patients (n=18) and healthy controls (n=24), by targeted MRM-MS. Bland-Altman plot comparing APCS mean concentration levels between LN cases, SLE cases and healthy controls (HC). The median serum APCS concentrations were determined as 7.325 (IQR: 4.365-9.605) and 7.24 (IQR: 5.23-9.47) and 8.255 (IQR: 7.155-9.57) μg/ml in the serum of healthy controls, SLE and LN, respectively. No statistically significant differences were observed between the three groups (p-value=0.51). Statistical analysis was performed using non-parametric tests. The three group analysis was performed using the Kruskal-Wallis rank test, while the two group analyses using the Wilcoxon rank-sum test (HC vs SLE, p-value=0.67; HC vs LN, p-value=0.30; SLE vs LN, p-value=0.37). Vertical lines present the median and interquartile range.

**Figure S6: Representative images of H+E staining of the validation set.**

(A-D) H+E images of kidney sections from C57/Bl6 (B6), NZW, MRL/J and BXSB.B6.Yaa (BXS) wild type mice, respectively. Normal glomerular capillaries (arrows) and tubules are shown. (E-G) H+E stained sections of lupus-prone B6.NZMSle123 (24 Weeks), NZB/W (28 Weeks) and MRL/lpr (12 Weeks) mice, respectively. Loss of glomerular capillaries and development of fibrosis (arrows) are seen. (H) H+E stained sections of lupus-prone BXSB.Yaa (16 Weeks), showing loss of glomerular capillaries and development of extensive fibrosis (arrows). Scale bar=100μm.

##
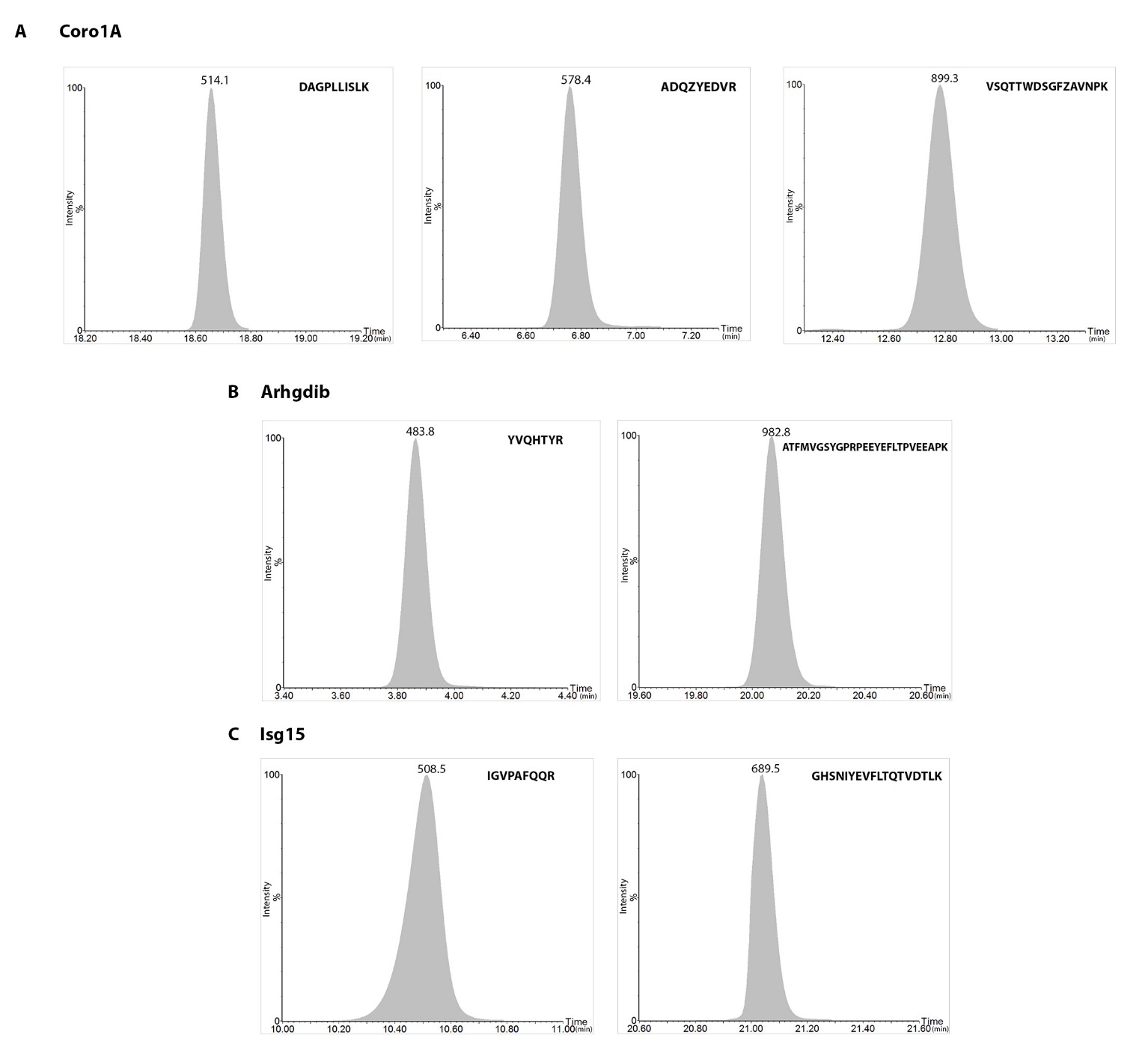
Figure S1

##
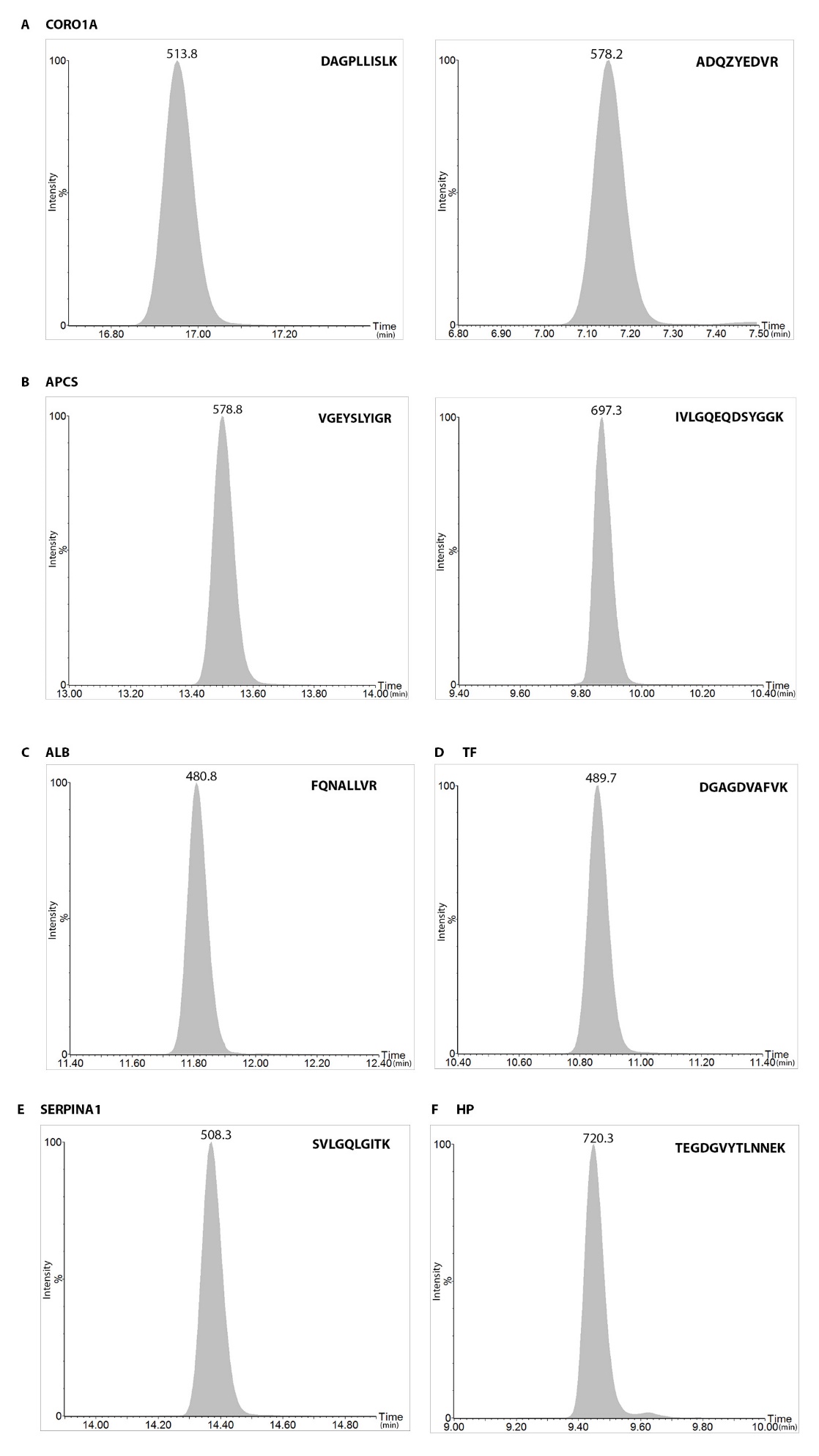
Figure S2

##
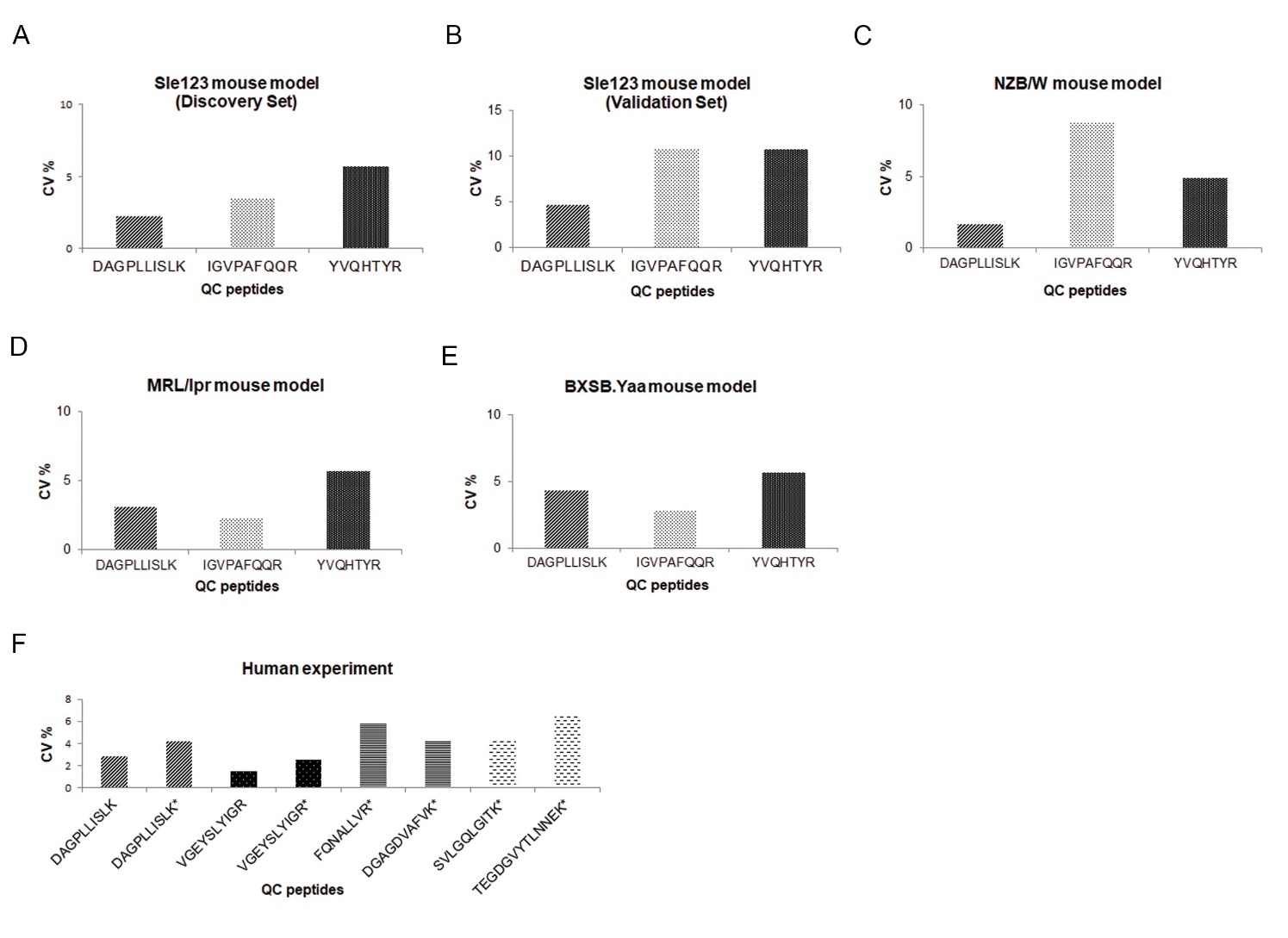
Figure S3

##
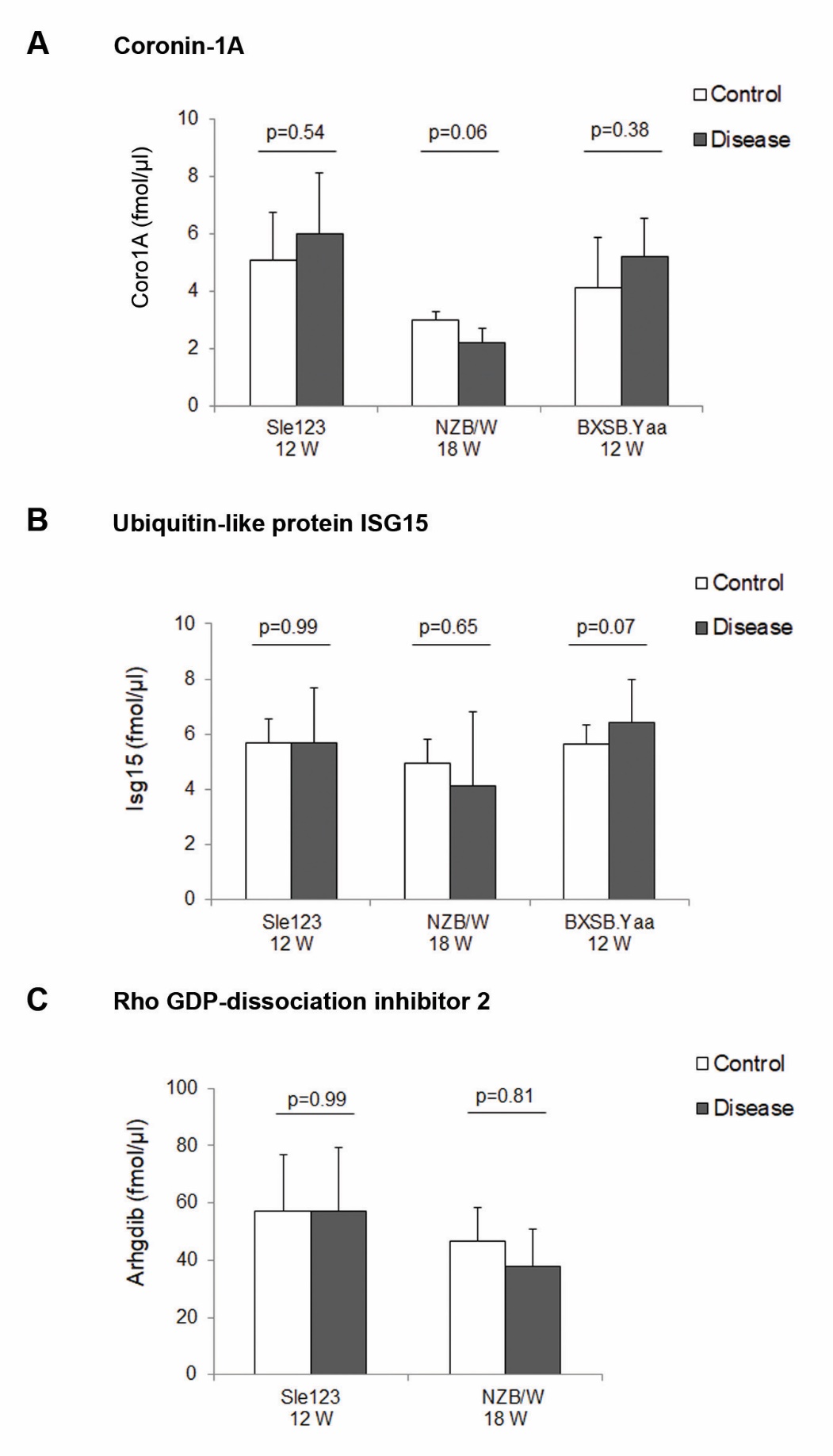
Figure S4

##
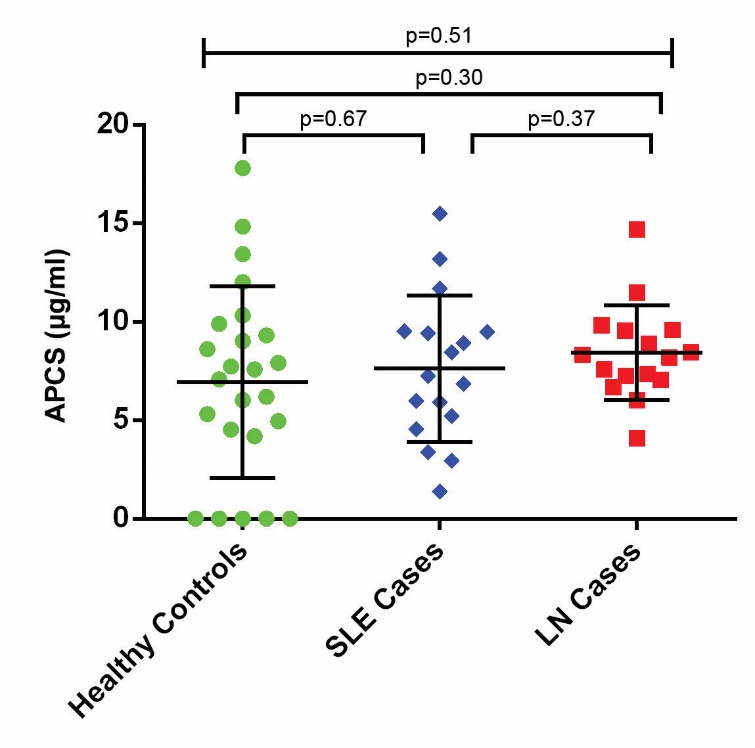


## **Figure S5**

##
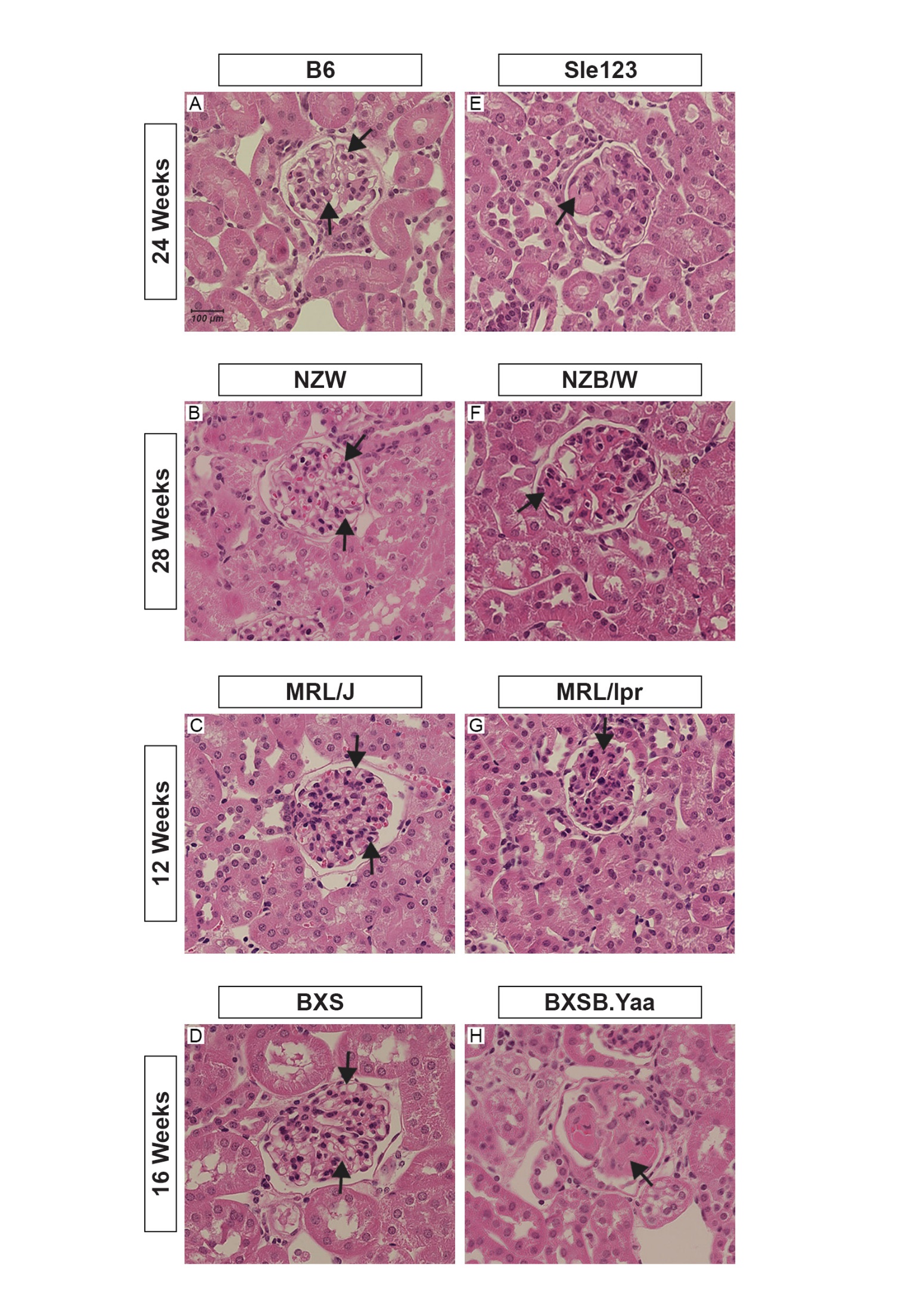
Figure **S6**
